# 5-Hydroxymethylfurfural and Isoverbascoside Alleviate Oxidative Damage INS-1 and MIN6 β-Cells by Activating Autophagy and Inhibiting Apoptosis

**DOI:** 10.3390/metabo16010048

**Published:** 2026-01-06

**Authors:** Xianglong Meng, Yuting Li, Xiang Han, Ziang Li, Zhulin Bu, Yuhui Wu, Xiaofen Li, Shuosheng Zhang, Yuting Dai

**Affiliations:** 1 College of Traditional Chinese Medicine and Food Engineering, Shanxi University of Chinese Medicine, Jinzhong 030619, China; 2 Shanxi Key Laboratory of Tradition Herbal Medicines Processing, Jinzhong 030619, China; 3 Traditional Chinese Medicine Processing Techniques Hertiage Base (Shanxi University of Chinese Medicine), National Adminstration of Traditional Chinese Medicine, Jinzhong 030619, China; 4 Key Research Laboratory of Processing and Innovation in Chinese Medicinal Materials, Shanxi University of Chinese Medicine, Jinzhong 030619, China; 5 First Clinical College, Shanxi University of Chinese Medicine, Jinzhong 030619, China

**Keywords:** T2DM, autophagy, apoptosis, pancreatic β cell, 5-Hydroxymethylfurfural, isoverbascoside

## Abstract

**Background/Objectives**: In type 2 diabetes (T2DM), dysregulated glucose and lipid metabolism impair cellular energy sensing and inhibit autophagy, leading to the accumulation of dysfunctional cellular components, increased inflammation and oxidative stress, and activation of the intrinsic apoptotic pathway. Prepared Rehmannia glutinosa is an anti-diabetic traditional Chinese medicine whose active monomers, including 5-Hydroxymethylfurfural (5-HMF) and isoverbascoside, exhibit potential antioxidant and anti-apoptotic effects. However, their role in β-cell protection remains unexplored. This study aims to investigate the protective mechanisms of 5-HMF and isoverbascoside against H_2_O_2_-induced oxidative damage in pancreatic β-cells. **Methods**: INS-1 and MIN6 β-cells were treated with 5-HMF and isoverbascoside (20 μM, 40μM) for 24 h under H_2_O_2_-induced oxidative stress. Multiple techniques were employed, including transcriptomics, proteomics, machine learning, Western blot analysis, and molecular docking. Flow cytometry and Hoechst 33342 staining were used to assess apoptosis, while autophagy was evaluated via LC3 fluorescence intensity and Beclin-1 expression. Chloroquine (CQ), an autophagy inhibitor, was applied to further examine autophagy’s role. **Conclusions**: 5-HMF and isoverbascoside enhance autophagic activity in pancreatic β-cells, attenuate oxidative stress-induced apoptosis, and improve cell survival and proliferation. These findings underscore their potential as protective agents in T2DM by modulating the autophagy–apoptosis balance.

## 1. Introduction

Type 1 diabetes mellitus (T1DM), type 2 diabetes mellitus (T2DM), and other subtypes of diabetes mellitus (DM) are several types of DM, a metabolic disease marked by chronic hyperglycemia [1,2]. Over 90% of the 537 million persons aged 20 to 79 who have diabetes, according to the International Diabetes Federation (IDF), have T2DM [3,4]. Predictions indicate that the number of T2DM cases will rise to 640 million by 2040, posing a substantial challenge to public health on a global scale [5]. The homeostasis of pancreatic β cells is crucial for insulin synthesis and secretion, and it is regulated by various internal and external factors, including autophagy, apoptosis, oxidative stress, nutritional status, inflammatory response, genetic factors, hormone levels, and physiological status. The presence of an abnormality in any link can lead to DM’s occurrence and development [6,7,8,9].

Autophagy is a diverse process that contributes to the maintenance of intracellular metabolic homeostasis by breaking down and reusing damaged organelles [10]. The extent of autophagy is of vital importance for preserving cellular homeostasis. Insufficient autophagy resulting from nutrient deficiency and aging, along with excessive autophagy triggered by increased reactive oxygen species (ROS), may contribute to the onset and progression of T2DM [11]. The complex regulation of autophagy is governed by a network of interconnected signaling pathways. Autophagy is precisely regulated by a complex signaling network that integrates nutritional, energetic, and stress signals. For example, under conditions of anabolic signaling activation such as by insulin and growth factors, or in response to cellular stressors including mitochondrial dysfunction and endoplasmic reticulum stress, the mammalian target of rapamycin complex 1 (mTORC1) pathway is activated, thereby suppressing autophagy initiation [12]. Similarly, the Phosphatidylinositol 3-Kinase/Ak strain transforming (PI3K/Akt) signaling pathway plays a significant inhibitory role in autophagy [13]. In contrast, AMP-activated protein kinase (AMPK), which functions as a cellular energy sensor, is activated under low-energy conditions and positively regulates autophagy through multiple mechanisms, including direct antagonism of mTORC1 function [14,15]. These core pathways, together with other stress-responsive signals (the JNK pathway), ultimately converge on the core autophagy initiation machinery, such as the Unc-51 Like Autophagy Activating Kinase 1 (ULK1) complex and the Beclin 1–Vps34 complex, to enable precise control of autophagic activity [16,17].

Apoptosis is delineated as the process by which cells undergo self-destruction through specific programmed mechanisms under both physiological and pathological circumstances [18]. In T2DM, apoptosis is regarded as a critical mechanism for the elimination of damaged or deleterious pancreatic β cells, which is indispensable for the preservation of overall health. Nevertheless, certain cytokines may stimulate lipid toxicity, induce immune system dysfunction, or enhance oxidative stress, resulting in excessive apoptosis of pancreatic β cells and disrupting their homeostasis, thereby facilitating the progression of DM [19]. T2DM can cause an increase in the levels of inflammatory factors such as tumor necrosis factor alpha (TNF-α), interleukin-6 (IL-6), and C-reactive protein (CRP) in the body. Among them, inflammatory factors can promote insulin resistance by activating pathways such as the nuclear factor kappa B (NF-κB) pathway. These inflammatory factors participate in the pathological mechanism of T2DM by promoting insulin resistance, damaging pancreatic β cell function, and interfering with normal metabolic processes. The Bcl-2 associated X protein (Bax) makes holes in the mitochondrial outer membrane, increasing its permeability and causing cytochrome c and other apoptosis-promoting substances to be released. This cascade activates Caspase-9, which subsequently activates Caspase-3, Caspase-7, and others, thereby promoting cellular apoptosis [20]. Furthermore, several signaling pathways play a role in controlling cell apoptosis, including Interleukin-1 β (IL-1β), NF-κB, p38 Mitogen-Activated Protein Kinase (p38MAPK), and c-Jun N-terminal Kinase signaling pathway (JNK). These pathways can induce cell apoptosis in response to diverse external stimuli through mitochondrial, death receptor, and endoplasmic reticulum pathways [21,22,23,24,25].

The equilibrium between autophagy and apoptosis holds paramount significance for the homeostatic state of pancreatic β cells, which is indispensable for the appropriate secretion of insulin. An imbalance between these two processes not only impairs the viability of β cells but also accelerates the advancement of T2DM [26]. As a consequence, the analysis of the interconnection between autophagy and apoptosis in pancreatic β cells, along with the impact of traditional Chinese medicines (TCM) and their formulations on T2DM treatment, has become a prominent research subject.

Rehmanniae Radix Praeparata (RRP), as a representative of medical and edible homology, is the steamed product of raw Rehmannia, which has been widely documented in classical books and has been employed for millennia in the management of diabetes [27,28,29]. Recent studies in the contemporary literature have substantiated that various chemical constituents present in RRP, notably 5-HMF and isoverbascoside, are implicated in the mitigation of diabetes through multiple mechanisms. These compounds, which have undergone extensive investigation, have been demonstrated to influence glucose regulation [27,30], possess antioxidant properties [31], reduce inflammation [32], and enhance energy metabolism [33]. Previous studies conducted by the research team have established that RRP and its constituent compounds possess the capacity to mitigate insulin resistance in key organs such as the liver, skeletal muscle, and fat tissue [34]. It remains unknown whether these compounds protect β-cells by regulating autophagy–apoptosis equilibrium under hyperglycemic oxidative stress, which constitutes the primary hypothesis to be tested in this study. Through integrated transcriptomic, proteomic, machine learning, and molecular biology analyses, it was demonstrated that these compounds enhanced pancreatic β-cell autophagy, reduced oxidative stress-induced apoptosis, and improved cell survival and proliferation. A protective role against T2DM progression was revealed by these findings, with evidence-based support being provided for the application of these herbal compounds in developing traditional medicine-inspired treatment strategies.

## 2. Materials and Methods

### 2.1. Cells

The rat islet cell carcinoma INS-1 and mouse islet beta cell line MIN6 were both purchased from Haixing Biotechnology Co., Ltd., Suzhou, China (item number: TCR-C608; TCM-C787). This study was conducted at the Key Laboratory of Chinese Medicinal Processing, Shanxi University of Chinese Medicine.

### 2.2. Drugs and Reagents

5-HMF (mass fraction ≥ 98%), isoverbascoside (MST-13122711, Shanghai Poetry Dan DE Standards Technical Services Co., Ltd., Shanghai, China) (mass fraction ≥ 98%); Chloroquine (HY-17589A), Rapamycin (HY-10219, MedChemexpress Biotechnology Inc., Morristown, NJ, USA) (purity: 99.94%); 30% hydrogen peroxide (the H_2_O_2_ working solution was freshly prepared in complete medium immediately before each experiment); RPMI1640 medium (10491), DMEM high-glucose medium (D5194, Beijing Mr. Lai treasure technology Co., Ltd., Beijing, China); fetal bovine serum (FBS, FCS500, Suzhou by Kordra Biotechnology Co., Ltd., Suzhou, China); 0.25% trypsin EDTA digestion solution (MA0232), penicillin streptomycin solution (MA0110, Dalian Mellon, Biological Technology Co., Ltd., Dalian, China); CCK-8 detection kit (C0038), EdU-488 cell proliferation detection kit (C0071S), BCA protein concentration determination kit (P0012), high-sensitivity ECL chemiluminescence kit (P0018S), 4% paraformaldehyde fixative (P0099), immunostaining permeabilization solution Triton X-100 (P0096), microfilament green fluorescent probe (C2201S) RIPA lysis buffer (P0013B), protease/phosphatase inhibitor (P1051, Shanghai Biyuntian Biotechnology Co., Ltd., Shanghai, China), Polyvinylidene Fluoride (PVDF) membrane, Beclin1 (A21191, dilution: 1:1000, Wuhan Aibotaike Biotechnology Co., Ltd., Wuhan, China), Bax (A19684, dilution: 1:1000, Wuhan Aibotaike Biotechnology Co., Ltd., Wuhan, China), Bcl-2 (A18415, dilution: 1:1000, Wuhan Aibotaike Biotechnology Co., Ltd., Wuhan, China), β-actin (AC038, dilution: 1:1000, Wuhan Aibotaike Biotechnology Co., Ltd., Wuhan, China), HRP goat anti rabbit secondary antibody (AS014, dilution: 1:1000, Wuhan Aibotaike Biotechnology Co., Ltd., Wuhan, China), Cy3 goat anti rabbit secondary antibody (AS007, dilution: 1:3000, Wuhan Aibotaike Biotechnology Co., Ltd., Wuhan, China).

### 2.3. Instruments

Fully automatic cell counter (Countess 3), cell culture box (3131-GP, Thermo Fisher Scientific Co., Ltd., Waltham, MA, USA); fluorescence inverted biological microscope (D-35578 Wetziar, Leica GmbH, Wetzlar, Germany); Gen 5 multifunctional microplate reader (Biotek, Winooski, VT, USA); gel imaging system (ChemiDoc XRS+, Bio Rad, Hercules, CA, USA); flow cytometer (NovoCyte 2060R, Agilent Technologies Co., Ltd., Santa Clara, CA, USA).

### 2.4. Cell Culture and Establishment of Oxidative Damage Model 

INS-1 cells were cultured in RPMI1640 medium containing 10% FBS, 1% streptomycin, and 0.05 mmol·L^−1^ β-mercaptoethanol at 37 °C and 5% CO_2_. MIN6 cells were cultured in DMEM high-glucose medium containing 10% FBS, 1% streptomycin, and 0.05 mmol·L^−1^ β-mercaptoethanol using the same cultivation method as described above. After being planted in 96-well plates at a density of 1 × 10^5^ cells/mL, both cell lines were incubated for 48 h. The cells were then divided into a control group and several treatment groups, each duplicated six times, and subjected to hydrogen peroxide (H_2_O_2_) concentrations ranging from 10 to 400 μM. The Cell Counting Kit-8 (CCK-8) test was used to evaluate cell viability after a 2 h exposure to H_2_O_2_, and microscopy was used to look for morphological changes. Based on the results of CCK-8, this study selected INS-1 cells at a concentration of 50 μmol·L^−1^ H_2_O_2_ and MIN6 cells at a concentration of 200 μmol·L^−1^ H_2_O_2_, which induced a decrease in cell survival rate of about 50%, for all subsequent experiments to create an oxidative damage model. Finding the ideal H_2_O_2_ concentration for creating an oxidative damage model was the goal.

### 2.5. Cell Viability Testing

INS-1 and MIN6 cells were grown with precision in the logarithmic phase and then placed at a density of 1 × 10^5^/mL in a 96-well plate for a period of 24 h. The cells were precisely categorized into a normal group and a drug group, where the latter included the 5-HMF and isoverbascoside treatment groups. Concentrations of 10, 20, 40, 80, 160, 320, and 640 μM were, respectively, administered to these groups, and six replicates were set for each. The CCK-8 method was employed to assess cell viability 24 h after drug treatment, aiming to find the drug concentration range that did not adversely affect normal cell viability. According to the results, concentrations of 20 and 40 μM of the two compounds were used for subsequent oxidative stress protection studies. To evaluate the protective effects of the compounds against H_2_O_2_-induced oxidative damage, a standardized pretreatment protocol was employed. Cells were first treated with either 5-HMF or isoverbascoside (at the selected non-cytotoxic concentrations) for 24 h. Subsequently, the culture medium was replaced, and cells were exposed to the optimal concentration of H_2_O_2_ (as determined in Section 2.4) for 2 h to induce oxidative injury. Following this, cell viability was assessed using the CCK-8 assay. This “24 h drug pretreatment followed by 2 h H_2_O_2_ challenge” protocol was established as the standard procedure for all subsequent experiments investigating protective mechanisms, including apoptosis analysis and Western blotting.

### 2.6. Transcriptomic Analysis

To elucidate the transcriptomic mechanisms underlying the drug-mediated protection, total RNA was extracted from INS-1 and MIN6 cells subjected to four distinct treatments for comparative analysis: the Normal group (untreated control), the H_2_O_2_ model group (exposed only to the optimal concentration of H_2_O_2_ for 2 h), the 5-HMF protection group (pretreated with a high concentration of 5-HMF followed by H_2_O_2_ exposure), and the isoverbascoside protection group (pretreated with a high concentration of isoverbascoside followed by H_2_O_2_ exposure). Cells from each group were collected immediately after the respective treatment regimen for subsequent RNA isolation and transcriptomic sequencing.

INS-1 and MIN6 cells were used to extract proteins and RNA with SDS-Detergent Buffer. RNA was purified with DNase and checked via agarose gel electrophoresis. mRNA was enriched using Oligo (dT) beads and fragmented. cDNA was synthesized from mRNA, purified, and processed for end repair, A-tail addition, adapter ligation, and PCR enrichment to create a cDNA library. Clean reads were obtained by filtering the sequencing data. The HISTA2 software was used to analyze and reconstruct transcripts and calculate gene expression levels. DESeq2 and Principal Component Analysis (PCA) found differentially expressed genes (DEGs) with |log2FC| > 0.5 and FDR < 0.05. The Kyoto Encyclopedia of Genes and Genomes (KEGG, http://www.genome.jp/, accessed on 28 March 2024) and Gene Ontology (GO, http://geneontology.org/, accessed on 29 March 2024) databases were used for functional annotation. Expression levels were indicated by Fragments Per Kilobase of Exon per Million mapped fragments (FPKM) values. The clusterProfiler and enrichplot tools were used to perform Gene Set Enrichment Analysis (GSEA), and ggplot2 was used to show the results.

### 2.7. Proteomic Analysis

Refer to Section 2.6 for specific groups Following the respective treatments, cells from each group were lysed for protein extraction and subsequent liquid chromatography–tandem mass spectrometry (LC-MS/MS) analysis.

INS-1 and MIN6 cells were lysed to extract proteins using SDT buffer. Proteins were quantified with the Bicinchoninic Acid (BCA) assay and hydrolyzed using Filter-Aided Sample Preparation (FASP). Peptides were desalted, freeze-dried, and resuspended for Liquid Chromatography–Mass Spectrometry (LC-MS) analysis. After high-performance liquid chromatography (HPLC) separation, samples were analyzed with a timsTOF Pro mass spectrometer, and data were processed using MaxQuant software (version 1.6.14). Assessed peptide ion scores and protein abundance ratios, confirming accurate and reliable identification with high-quality data. Differentially expressed proteins (DEPs) were identified with Fold Change (FC) > 1.5 and *p* < 0.05. Hierarchical clustering analysis was performed using Cluster 3.0 and Java Treeview to create volcano and heat maps. Differentially expressed proteins (DEPs) were annotated for GO functions and KEGG pathways using Blast2GO (https://www.blast2go.com, accessed on 29 March 2024) and the KEGG database.

### 2.8. Protein Interaction Network Analysis

STRING (http://string-db.org/, accessed on 30 March 2024) was utilized to search for both direct and indirect interactions among target proteins in the database, and CytoScape software (version 3.2.1) was employed to create and examine the interaction network.

### 2.9. Matching Analysis Methods

The DEGs identified via transcriptomics were aligned with the quantitative results obtained from proteomics and compared based on the identifiers of genes or proteins. The Pearson correlation coefficient was applied to determine the connection between gene and protein expression, with volcano and heat maps being generated. Annotation of the GO functions and KEGG pathways of the shared differentially expressed factors was carried out using the Blast2GO and KEGG pathway databases.

### 2.10. Least Absolute Shrinkage and Selection Operator (LASSO) Screening for Differentially Expressed Energy Metabolism-Related Genes (EMRGs) and Molecular Docking

The LASSO algorithm, a method grounded in linear regression modeling, was employed for variable selection and compression, as well as for the acquisition of parameter estimates. This approach significantly diminishes the dimensionality of the feature space, effectively mitigates issues of overfitting and multicollinearity, and is extensively utilized and advocated within the field. LASSO regression is applied to identify diagnostic markers among differentially expressed genes. The 3D structures of 5-HMF and isoverbascoside were obtained from the chemical molecular database PubChem (https://pubchem.ncbi.nlm.nih.gov/, accessed on 25 June 2024). The gene protein entry numbers were queried from the protein database UniProt (https://www.uniprot.org/, accessed on 25 June 2024), and the 3D structures of the protein receptors corresponding to key genes were downloaded from the RCSB Protein Data Bank (PDB) (https://www.rcsb.org/, accessed on 25 June 2024). AutoDock Tools-1.5.6 software was used to perform molecular docking of 5-HMF and isoverbascoside with key genes Abelson Interactor 1 (ABI1)/7LXE, Immunoglobulin Heavy Constant Mu (IGHM)/3C2S, Interleukin-2 Receptor Subunit Gamma (IL2RG)/7S2R, Mannosyl-Oligosaccharide Glucosidase (MOGS)/5MHF, Cysteine Desulfurase (NFS1)/8PK8 as well as autophagy and apoptosis-related protein receptors B-cell lymphoma 2 (BCL-2)/2NL9, Mechanistic Target of Rapamycin (mTOR)/3KZ7, and Beclin 1/5YR0. LASSO regression was conducted on EMRGs, and partial likelihood bias was applied with 10-fold cross-validation to determine the optimal lambda (λ). When the mean square error was minimized, the accuracy of the prediction model in describing experimental data increased, and the corresponding λ value determined the degree of LASSO regression complexity adjustment.

### 2.11. Cell Apoptosis Detection

The cells were classified into four distinct groups—a normal group (Normal), a model group (Control), and high- and low-concentration drug groups—all of which were treated with H_2_O_2_. Following the instructions of the Annexin V Fluorescein Isothiocyanate Propidium Iodide (Annexin V-FITC/PI) staining kit, cells were digested with 0.25% EDTA-free trypsin, washed with Phosphate-Buffered Saline (PBS), and incubated in the dark with dye and buffer. The analysis of apoptosis was performed using the specified machine.

### 2.12. Cell Immunofluorescence Analysis

Without H_2_O_2_ treatment, the cells were divided into three groups: normal, model, and high- and low-concentration drug groups. After being gathered, cells were cleaned with PBS. After 20 min of fixation in 4% paraformaldehyde, cells were permeabilized for 15 min using Triton X-100 and blocked for an hour using 2% BSA. They were then treated for two hours with an LC3B primary antibody (1:200), followed by one hour in the dark with a green fluorescent probe (1:100) and a Cy3 secondary antibody (1:500). Nuclear staining was performed using 4′,6-diamidino-2-phenylindole (DAPI) following Tris-Buffered Saline with Tween 2 (TBST) washing. Fluorescence microscopy and Image J software (version 1.54) were used for imaging and quantifying fluorescence intensity.

### 2.13. Autophagy and Apoptosis-Related Protein Expression in Cells

Western blot analysis was performed to first confirm the protective effects of 5-HMF and isoverbascoside against H_2_O_2_-induced oxidative damage and then to investigate whether the observed anti-apoptosis was mediated through the autophagy pathway. Accordingly, INS-1 and MIN6 cells were assigned to several comparative groups for protein extraction: a Normal group (untreated control), a Model group (H_2_O_2_ only), Drug Protection groups (pretreated with low or high doses of 5-HMF or isoverbascoside followed by H_2_O_2_), and key mechanistic groups including a CQ + Model group (co-treated with the autophagy inhibitor Chloroquine and H_2_O_2_ to verify that autophagy blockade exacerbates apoptosis) and CQ + Drug Protection groups (co-treated with CQ, drug, and H_2_O_2_ to test if inhibiting autophagy reverses the protective effect). After discarding the culture medium, PBS was used to wash the cells. The cells were lysed on ice for half an hour using RIPA lysis buffer that contained protease and phosphatase inhibitors. The total protein from the cells was then extracted by centrifuging at 12,000 r·min^−1^ for 30 min at 4 °C and collecting the supernatant. The BCA protein concentration assay kit’s instructions were followed in order to determine the sample’s total protein concentration. Following protein denaturation, the material underwent 10% SDS-PAGE gel electrophoresis before being wet transferred at 250 mA for 1 h on a PVDF membrane. After sealing with 5% skim milk powder, the primary antibody solution, diluted 1:1000, was added, and the mixture was incubated. The membrane was washed three times with TBST, and then the secondary antibody solution (1:3000 dilution) was added for 1 h incubation. After the membrane was washed, Enhanced Chemiluminescence (ECL) solution was administered, and protein bands were captured using a gel imager. With β-actin as an internal reference, the relative expression levels of Beclin1, Bax, and Bcl-2 proteins in each group were analyzed using Image J software.

### 2.14. Statistical Analysis

Using GraphPad Prism 8.0, statistical analyses were carried out, and the outcomes were expressed as mean ± standard deviation. One-way ANOVA and *t*-tests were used to assess group differences, with significance marked by *p* < 0.05.

## 3. Results

### 3.1. The Effect of H_2_O_2_ on the Viability of Pancreatic β Cells

**Figure 1:**
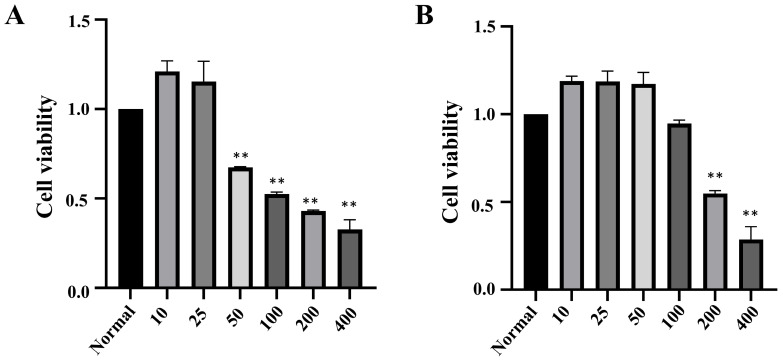
The effect of H_2_O_2_ on the survival rate of INS-1 and MIN6 cells (*n* = 3, ** *p* < 0.01). Note: (**A**) survival of INS-1 cell; (**B**) survival of MIN6 cells.

To establish a model of H_2_O_2_-induced cellular oxidative damage and determine the appropriate concentration for inducing such damage, INS-1 and MIN6 cells were treated with a gradient of H_2_O_2_ concentrations (10, 25, 50, 100, 200, 400 μmol·L^−1^) for 2 h. The activity of INS-1 cells increased at H_2_O_2_ concentrations of 10 μmol·L^−1^ and 25 μmol·L^−1^ but gradually decreased with further increase in concentration; MIN6 cells showed an increase in activity within the concentration range of 10 μmol·L^−1^ to 50 μmol·L^−1^, followed by a decrease in activity at higher concentrations. To establish a model of moderate oxidative damage, the concentrations selected were primarily based on inducing a substantial yet sub-lethal reduction in cell viability to approximately 50–70%. Consequently, 50 μmol·L^−1^ H_2_O_2_ was chosen for INS-1 cells (resulting in 67.13% viability) and 200 μmol·L^−1^ for MIN6 cells (resulting in 54.75% viability). Microscopic examination revealed characteristic morphological changes indicative of cellular damage following treatment with the selected H_2_O_2_ concentrations, including cell rounding, increased refractility, widening of intercellular spaces, and the appearance of features such as cellular shrinkage and membrane blebbing, which are associated with the apoptotic process (Figure 1).

### 3.2. Protective Effects of 5-HMF and Isoverbascoside on H_2_O_2_-Induced Damage in Pancreatic β Cells

INS-1 and MIN6 cells underwent a 24 h exposure to a range of concentrations of 5-HMF and isoverbascoside. The results showed that when the concentration of the treatment group reached 640 μmol·L^−1^, the viability of both types of cells decreased, while lower concentrations (10, 20, 40, 80, 160, 320 μmol·L^−1^) had no significant effect on cell viability. Therefore, in subsequent studies, 5-HMF and isoverbascoside were selected for administration at concentrations not exceeding 320 μmol·L^−1^.

**Figure 2:**
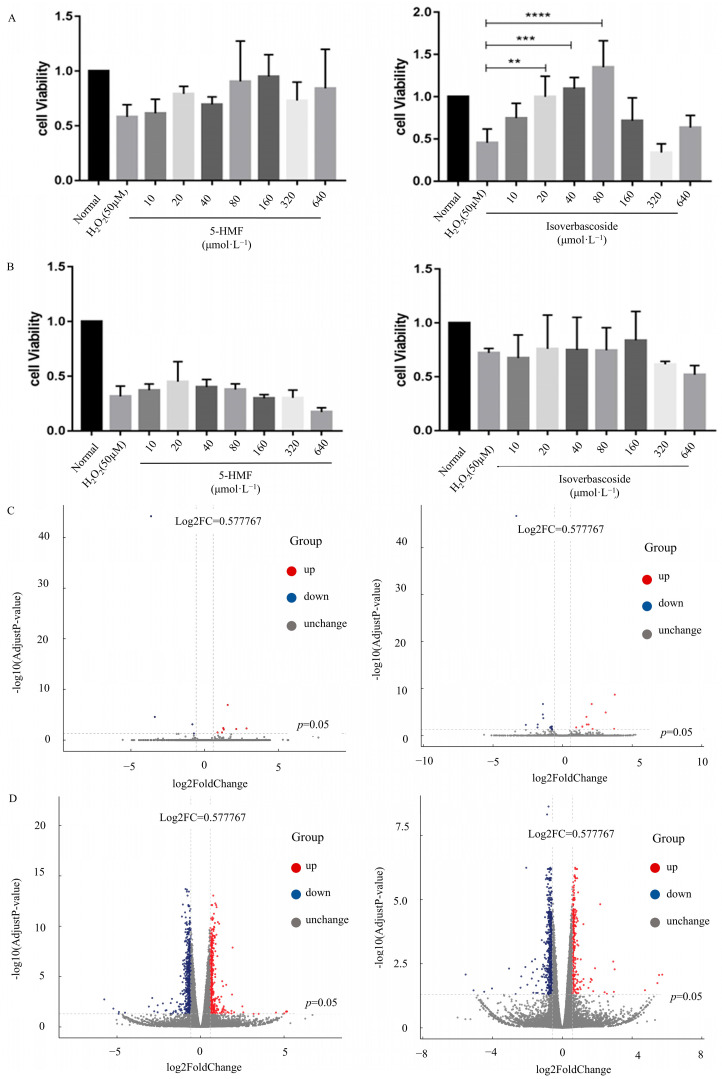
The effects of 5-HMF, isoverbascoside on the survival rate of INS-1 and MIN6 cells induced by H_2_O_2_ (*n* = 3, ** *p* < 0.01, *** *p* < 0.001, **** *p* < 0.0001). Note: (**A**): survival of INS-1 cell; (**B**): survival of MIN6 cells; (**C**): volcanic map of differentially expressed genes in INS-1 cells; (**D**): volcanic map of differentially expressed genes in MIN6 cells.

A stable H_2_O_2_ damage model was established, and safe doses were determined before further studying the protective effects of 5-HMF and isoverbascoside on cells. After 24 h of intervention with 5-HMF and isoverbascoside at concentrations of 10, 20, 40, 80, 160, 320, and 640 μ mol·L^−1^ in two cell types, H_2_O_2_ was subsequently applied to induce cell damage for 2 h. Compared with the model group, the cell survival rates of both cell types showed an upward trend after treatment with compounds of different concentrations. It is worth noting that treatment with isoverbascoside at concentrations of 20 μmol·L^−1^ (*p* < 0.01), 40 μmol·L^−1^ (*p* < 0.001), and 80 μmol·L^−1^ (*p* < 0.0001) in INS cells significantly improved cell survival rate, with cell survival rates of 90.51%, 94.39%, and 97.61%, respectively. A protective trend was also observed for 5-HMF in INS-1 cells, although statistical significance was not reached at the tested concentrations. Similarly, in MIN6 cells, pretreatment with both compounds demonstrated a concentration-dependent increase in cell survival against H_2_O_2_-induced damage, confirming their protective effects. Based on these results, the maximum safe and effective concentration for subsequent mechanistic assays was determined to be 320 μmol·L^−1^ (Figure 2).

### 3.3. Differentially Expressed Genes (DEGs) Analysis in Transcriptomic

**Figure 3:**
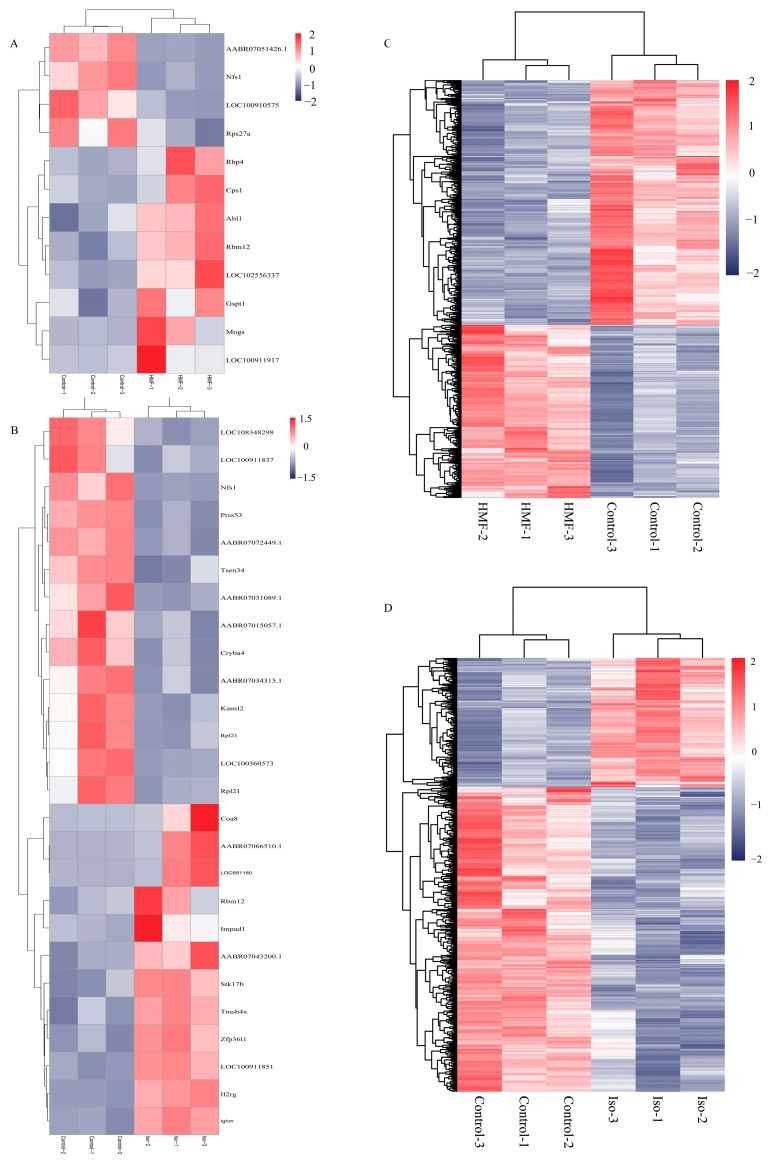
Transcriptomic data analysis of oxidative damage. Note: (**A**): differential gene Control vs. 5-HMF in INS-1 cells; (**B**): differential gene Control vs. Isoverbascoside in INS-1 cells; (**C**): differential gene Control vs. 5-HMF in MIN6 cells; (**D**): differential gene Control vs. Isoverbascoside in MIN6 cells.

Employing a |log2FC| > 0.5 and FDR < 0.05 as the guiding criteria, harnessing the power of the negative binomial distribution (DESeq2) and subsequently validated through the empirical scrutiny of digital gene expression data within the R environment (edgeR). This comprehensive investigation spanned across the Normal group, Control group, 5-HMF group, and isoverbascoside group. In the INS-1 cells, transcriptome sequencing highlighted 277 DEGs from the comparison between the Control and 5-HMF groups. Furthermore, an impressive 485 DEGs were discerned in the contrast between the Control group and the isoverbascoside group. In MIN6 cells, a remarkable 1503 DEGs were found when comparing the Control group to the 5-HMF group, whereas 1318 DEGs were detected in the comparison between the Control group and the isoverbascoside group (Table 1). Detailed information on DEGs is provided in the transcriptomic Supplement Tables S1–S8 (Figure 3).

### 3.4. GO Functional Analysis in Transcriptomics

GO functional enrichment analysis found that in INS-1 cells, there was a significant enrichment in ATP-dependent activity and DNA-related functions in the Control group compared to the Normal group. In contrast, the 5-HMF group exhibited enrichment in carbamoyl phosphate synthase (ammonia) activity relative to the control group, while the isoverbascoside group was predominantly enriched in the cellular response to raffinose. In MIN6 cells, differential gene expression in the Control group was primarily enriched in processes associated with cellular macromolecular metabolism, cellular metabolism, cellular protein metabolism, and cellular nitrogen compound metabolism relative to the Normal group. The 5-HMF group was chiefly enriched in binding processes related to nucleic acid binding, heterocyclic compound binding, protein folding chaperone activity, organic cytochrome compound binding, and RNA binding compared to the control group. Meanwhile, the isoverbascoside group demonstrated enrichment in binding processes, including RNA binding, nucleic acid binding, heterocyclic compound binding, organic cyclic compound binding, pure ribonucleoside triphosphate binding, and ATP binding, as well as metabolic processes such as cellular metabolism, cellular macromolecular metabolism, and macromolecular metabolism.

**Figure 4:**
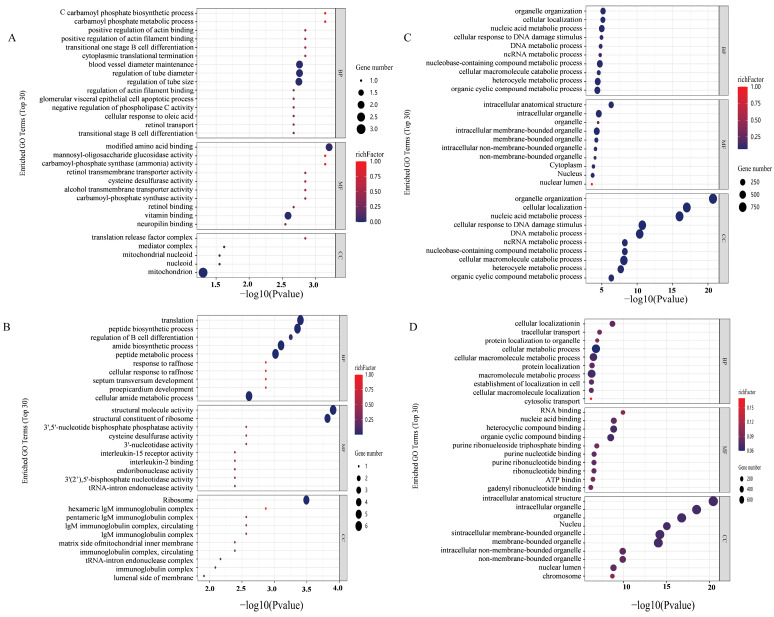
Transcriptomic data analysis of oxidative damage. Note: (**A**): GO functional enrichment map of Control vs. 5-HMF in INS-1 cellss; (**B**): GO functional enrichment map of Control vs. Isoverbascpside in INS-1cells; (**C**): GO functional enrichment map of Control vs. 5-HMF in MIN6 cells; (**D**): GO functional enrichment map of Control vs. Isoverbascpside in MIN6 cells.

These findings suggest that 5-HMF and isoverbascoside primarily influence processes related to DNA activity, sucrose response, ribosome function, and catabolism, thereby impacting T2DM through the regulation of associated autophagy pathways, activation of apoptotic signaling, and modulation of cellular energy metabolism (Figure 4).

### 3.5. KEGG Pathway Analysis in Transcriptomics

KEGG pathway enrichment analysis showed that in INS-1 cells, compared with the control group, the 5-HMF group had a significant bias towards metabolic pathways, including the complex network of thiamine metabolism, nitrogen metabolism, alanine, aspartate, and glutamate metabolism, as well as autophagy processes in animals. Conversely, the isoverbascoside group predominantly gravitated towards pathways such as Thiamine metabolism, the JAK-STAT signaling pathway, and the PI3K Akt signaling pathway. In the realm of MIN6 cells, the 5-HMF group exhibited a marked focus on Mitophagy in animals, the multifaceted process of apoptosis across various species, the pivotal p53 signaling pathway, and the dynamics of autophagy in animals, in contrast to the Control group. Meanwhile, the isoverbascoside group primarily underscored mitophagy in animals, autophagy in animals, and the IL-17 signaling pathway.

**Figure 5:**
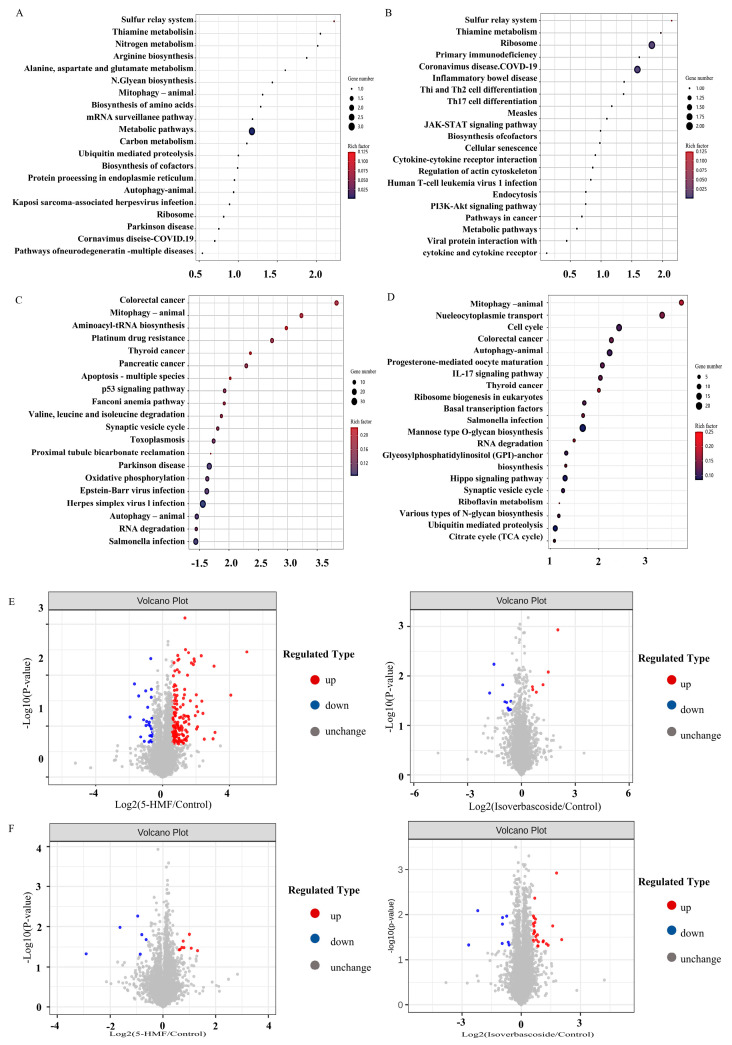
Transcriptomic data analysis of oxidative damage in INS-1 cells and MIN6 cells. Note: (**A**): KEGG pathway enrichment map of Control vs. 5-HMF in INS-1 cells; (**B**): KEGG pathway enrichment map of Control vs. Isoverbascpside in INS-1 cells; (**C**): KEGG pathway enrichment map of Control vs. 5-HMF in MIN6 cells; (**D**): KEGG pathway enrichment map of Control vs. Isoverbascpside in MIN6 cells; (**E**): volcano diagram of differentially expressed proteins in INS-1 cells; (**F**): volcano diagram of differentially expressed proteins in MIN6 cells.

These revelations suggested that both 5-HMF and isoverbascoside exert a significant influence on T2DM through their modulation of metabolic pathways, including nitrogen metabolism, thiamine metabolism, and the metabolism of alanine, aspartate, and glutamate, as well as through critical signaling pathways such as JAK-STAT, PI3K Akt, IL-17, autophagy, and cell apoptosis. Their regulatory effects on insulin secretion and sensitivity, oxidative damage, autophagy, and cell apoptosis subsequently shape the trajectory of T2DM (Figure 5).

### 3.6. Differentially Expressed Proteins (DEPs) Analysis in Proteomic

**Figure 6:**
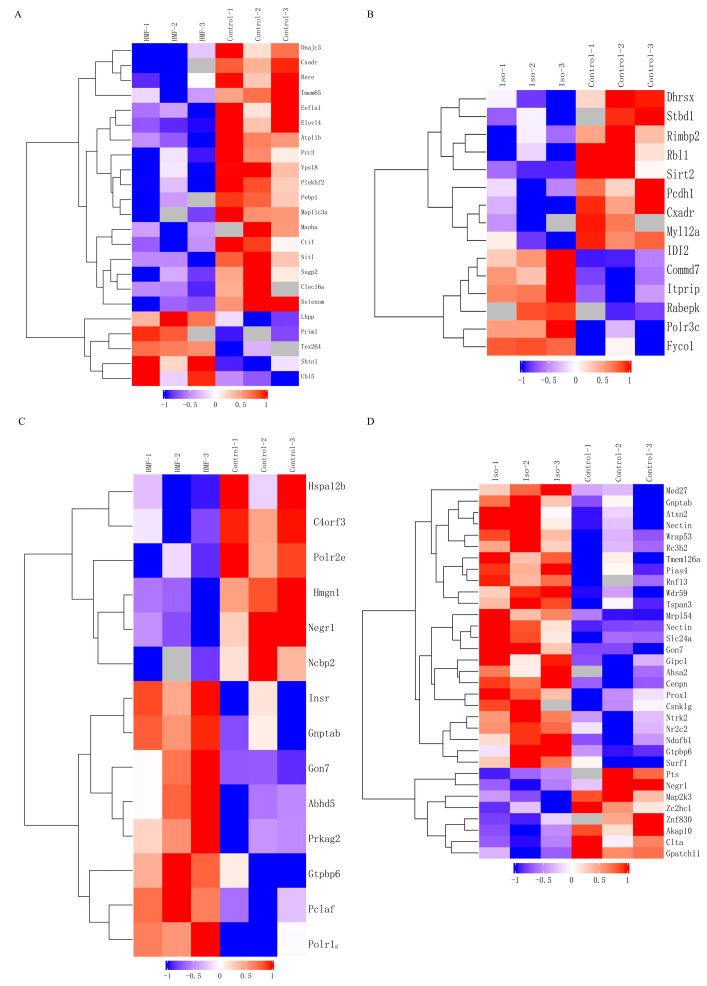
Transcriptomic data analysis of oxidative damage in INS-1 cells and MIN6 cells. Note: (**A**): Differential Gene-Related Heatmap of Control vs. 5-HMF in INS-1 cells; (**B**): Differential Gene-Related Heatmap of Control vs. Isoverbascpside in INS-1 cells; (**C**): Differential Gene-Related Heatmap of Control vs. 5-HMF in MIN6 cells; (**D**): Differential Gene-Related Heatmap of Control vs. Isoverbascpside in MIN6 cells.

The results indicated that, in INS-1 cells, the isoverbascoside group identified 8 down-regulated proteins, while the 5-HMF group identified 6 proteins (3 up-regulated, 3 down-regulated) compared to the Control group. In MIN6 cells, the isoverbascoside group identified 5 proteins (3 up-regulated, 2 down-regulated), and the 5-HMF group identified 9 proteins (7 up-regulated, 2 down-regulated) (Figure 5). Cluster analysis was performed on these data, resulting in differential expression heatmaps shown in Figure 6 and Table 1. Detailed information on DEPs is provided in the proteomic.

### 3.7. GO Functional Analysis in Proteomic

In INS-1 cells, the 5-HMF group is enriched in biological processes such as glucocorticoid secretion, the Netrin activation signaling pathway, the mannan metabolic process, and the catabolic processes of cell wall macromolecules, when compared to the Control group. In contrast, the isoverbascoside group demonstrated primary involvement in the organization of cellular junctions, the binding of type 1 to type 2 angiotensin receptors, and various other molecular functions, relative to the Control group. The 5-HMF group distinctly emphasized biological processes related to the biosynthesis, metabolism, and transport of cholesterol and steroids, as well as molecular functions associated with cholesterol and sterol protein activity, compared to the isoverbascoside group.

**Figure 7:**
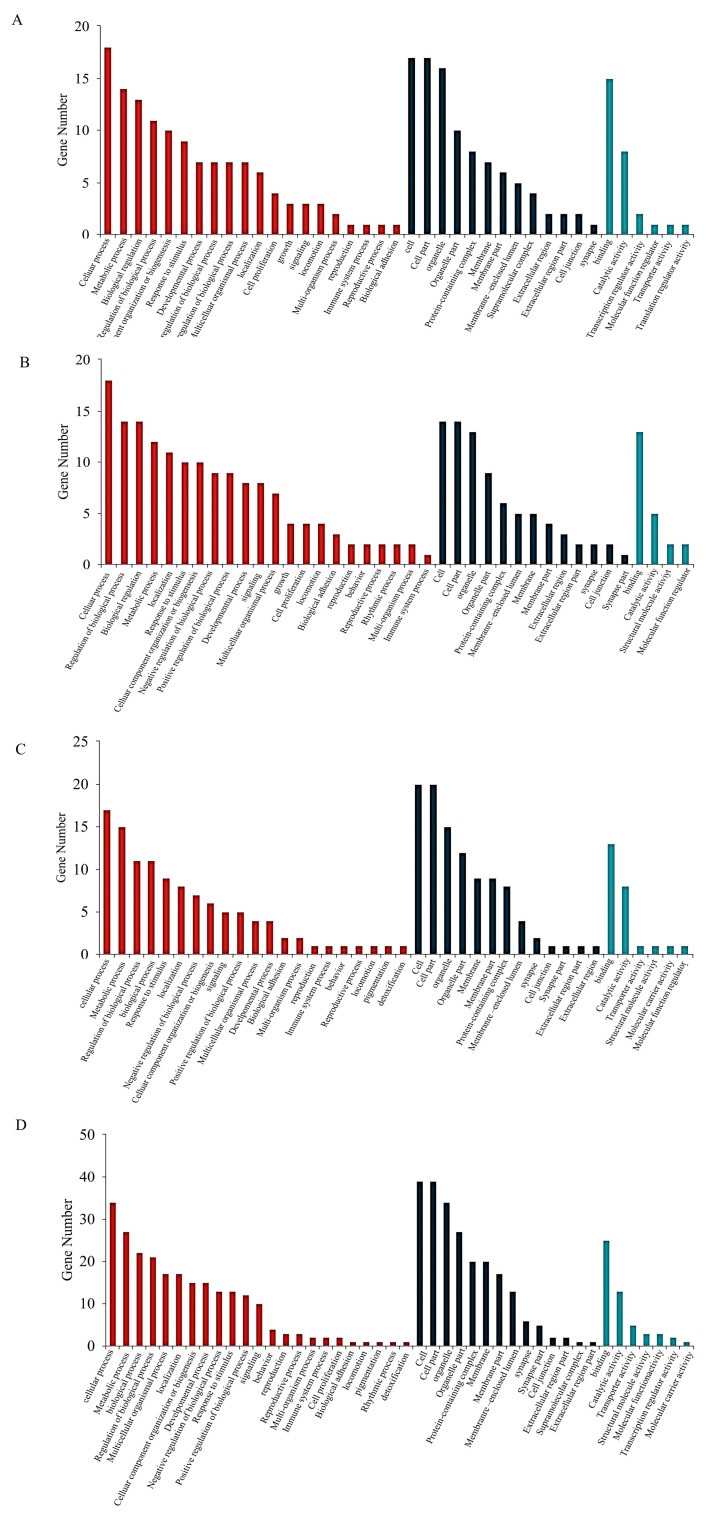
Transcriptomic data analysis of oxidative damage in INS-1 cells and MIN6 cells. (**A**): GO function enrichment of Control vs. 5-HMF in INS-1 cells; (**B**): GO function enrichment of Control vs. Isoverbascoside in INS-1 cells; (**C**): GO function enrichment of Control vs. 5-HMF in MIN6 cells; (**D**): GO function enrichment of Control vs. Isoverbascoside in MIN6 cells.

Turning to MIN6 cells, the differentially expressed proteins within the 5-HMF group, when compared to the Control group, predominantly engage in molecular functions such as the regulation of glycolytic processes, the formation of transferase complexes, RNA polymerase complexes, and the binding of 3-phosphoinositide-dependent protein kinases. Conversely, the differentially expressed proteins in the isoverbascoside group, when contrasted with the Control group, primarily pertain to molecular functions such as integral components of the plasma membrane, intrinsic components of the plasma membrane, and protein tyrosine kinase activity, among others. Furthermore, the differentially expressed proteins in the 5-HMF group, when compared to the isoverbascoside group, predominantly involve molecular functions such as insulin-like growth factor receptor binding and growth factor activity (Figure 7).

### 3.8. KEGG Pathway Analysis in Proteomic

**Figure 8:**
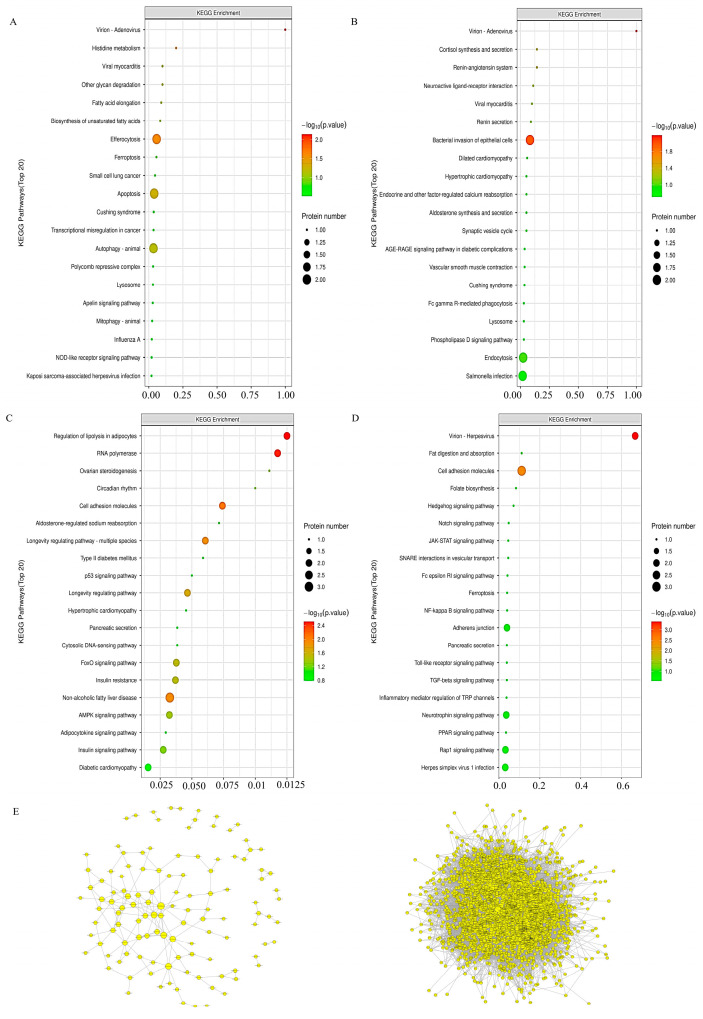
Proteomic data analysis of oxidative damage in INS-1 cells and MIN6 cells. Note: (**A**): KEGG pathway enrichment of Control vs. 5-HMF in INS-1 cells; (**B**): KEGG pathway enrichment of Control vs. Isoverbascoside in INS-1 cells; (**C**): KEGG pathway enrichment of Control vs. 5-HMF in MIN6 cells; (**D**): KEGG pathway enrichment of Control vs. Isoverbascoside in MIN6 cells; (**E**): construction of PPI network in INS-1 cells and MIN6 cells.

In INS-1 cells, a comparative analysis unfolded, revealing that, in stark contrast to the Control group, the pathway associated with the 5HMF group predominantly accentuated the intricate processes of autophagy in animals, efferocytosis, and apoptosis, among others. When placed alongside the 5HMF group, the isoverbascoside group primarily directed its focus towards the biosynthesis of unsaturated fatty acids, the phenomenon of apoptosis, lipid metabolism, and the complex dynamics of atherosclerosis. Shifting our gaze to MIN6 cells, a similar comparative lens unveiled that the pathway of the isoverbascoside group, in juxtaposition with the Control, chiefly illuminated fat digestion and absorption, alongside folate biosynthesis, whereas the pathway of the 5-HMF group was largely centered upon cell adhesion molecules. Notably, in comparison to the isoverbascoside group, the 5-HMF group also predominantly emphasized cell adhesion molecules (Figure 8).

### 3.9. Protein–Protein Interaction (PPI) Network Construction

To elucidate the protein interactions underlying the observed effects, protein–protein interaction (PPI) networks were constructed separately for INS-1 and MIN6 cells using the STRING database, based on the differentially expressed proteins identified from the proteomic analysis. Hub proteins within each network were identified by topological analysis of connectivity using CytoHubba in Cytoscape. In INS-1 cells, Gmps showed the highest connectivity, located at the center of the PPI network, followed by EBNA1 binding protein 2. In MIN6 cells, Gapdh showed the highest connectivity, located at the center of the PPI network, and exhibited downregulated expression (Figure 8).

### 3.10. Matching Analysis Methods

Conducted transcriptome and proteomic integration analysis in INS-1 cells and MIN6 cells subjected to oxidative stress damage treated with 5-HMF and isoverbascoside, respectively. The results showed that the two compounds exhibited commonalities and characteristic regulatory patterns at the molecular level. Compared with the Control group, treatment with 5-HMF jointly affected a series of pathways closely related to oxidative stress response and cellular homeostasis regulation at the dual omics level. These pathways focus on programmed cell death mediated by hydrogen peroxide and its positive regulation, Cdc42 protein signaling transduction, membrane transport mediated by Syntaxin and SNARE binding, positive regulation of reactive oxygen species and oxidative stress response, as well as biological processes such as epithelial structure maintenance. In contrast, treatment with isoverbascoside specifically accumulates in biological processes responsive to salt stress. The KEGG pathway enrichment results showed significant differences in the molecular mechanisms of action between the two compounds. Compared with the Control group, the core pathways jointly affected by 5-HMF treatment at the transcriptional and protein levels include autophagy in animal cells, mitochondrial autophagy, protein processing in the endoplasmic reticulum, Kaposi’s sarcoma associated herpesvirus infection, and pathways in various neurodegenerative diseases. In contrast, treatment with isoverbascoside accumulates in different sets of pathways at the same omics level, mainly including endocytosis and regulation of actin cytoskeleton. This analysis indicates that in response to oxidative stress, pentahydroxymethylfurfural mainly affects molecular networks related to cellular autophagy and protein quality control; the role of different verbascosides is more focused on regulating pathways related to cellular endocytosis and cytoskeleton remodeling.

In MIN6 cells, the GO functional enrichment results showed that 5-HMF and isoverbascoside have different molecular action characteristics. Compared with the Control group, the 5-HMF treatment group was significantly enriched in functions related to DNA damage response and nucleic acid metabolism regulation at both transcriptional and protein levels, mainly including cellular responses to DNA damage stimuli, positive regulation of cross damaged DNA synthesis and mRNA metabolism processes, and the breakdown metabolism of nitrogen-containing and heterocyclic compounds in cells. The group treated with isoverbascoside showed specific effects on the membrane structure and kinase activity of organelles, with their co enriched functional items concentrated in membrane structures such as lysosomal membranes/vacuolar membranes, as well as protein serine/threonine/tyrosine kinase activity. The analysis results indicate that in response to oxidative stress, pentahydroxymethylfurfural mainly intervenes in DNA damage response and metabolic reprogramming processes, while the effect of verbascoside is more concentrated in the organelle membrane system and kinase signaling network. The enrichment results of KEGG pathway showed that both compounds could significantly regulate the signal network closely related to the pathology and cell homeostasis of T2DM, but their action spectrum was significantly different. Compared with the Control group, the pathway core enriched by 5-HMF treatment at both omics levels is focused on energy metabolism and stress survival regulation, mainly including insulin signaling pathway, insulin resistance, AMPK signaling pathway, FoxO signaling pathway, autophagy, p53 signaling pathway, mTOR signaling pathway, PI3K Akt signaling pathway, MAPK signaling pathway, and oxidative phosphorylation. The treatment with isoverbascoside is enriched in another set of characteristic pathways, whose core involves cell membrane transport, inflammatory immune regulation, and catabolism, mainly including SNARE vesicle transport, lysosomes, autophagy, mTOR signaling pathway, MAPK signaling pathway, PI3K Akt signaling pathway, and inflammation-related pathways such as JAK-STAT, NF-κ B, and TNF signaling pathways. Please refer to Supplementary Tables S1–S8 for details.

**Figure 9:**
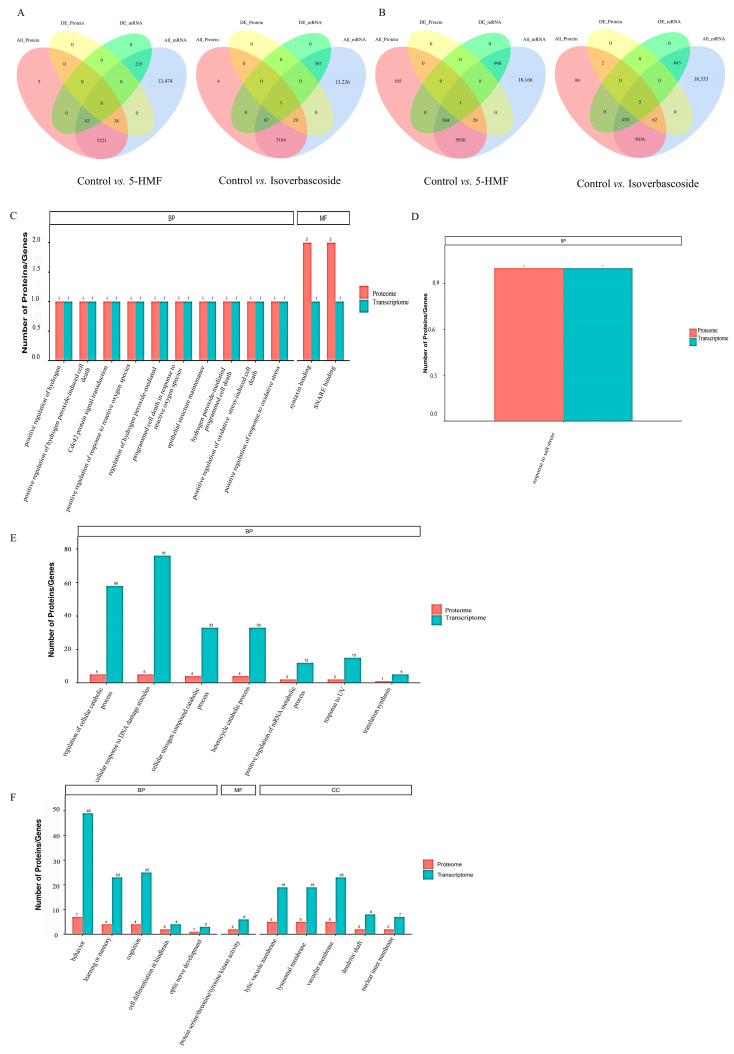
Combined analysis of transcriptomic and proteomic data on oxidative damage in INS-1 cells and MIN6 cells. Note: (**A**): Venn diagram of differentially expressed genes and proteins in INS-1 cells; (**B**): Venn diagram of differentially expressed gene proteins in MIN6 cells; (**C**): differentially expressed genes and protein GO functions enrichment of Control vs. 5-HMF in INS-1 cells; (**D**): differentially expressed genes and protein GO functions enrichment of Control vs. Isoverbascoside in INS-1 cells; (**E**): differentially expressed genes and protein GO functions enrichment of Control vs. 5-HMF in MIN6 cells; (**F**): differentially expressed genes and protein GO functions enrichment of Control vs. Isoverbascoside in MIN6 cells.

It is worth noting that research has found that genes enriched in GO function include CASP3 (apoptotic actuator) and ATM (DNA damage/autophagy regulatory center), as well as multiple genes that play a central role in oxidative stress (such as Abl1), lysosomal function (such as TFEB), and DNA damage response. The genes enriched in the KEGG pathway include key cell fate determining factors such as MAP1LC3A (autophagosome formation marker) and CASP3 (apoptosis executor), as well as genes that play a central role in DNA damage response (such as ATM), lysosomal function (such as TFEB), and neurodegenerative regulation (such as LRRK2). These central genes exhibit significant enrichment at both transcriptional and proteomic levels, collectively indicating deep interweaving and synergistic activation of oxidative stress-induced cell death and lysosome/autophagy pathways (Figure 9).

### 3.11. Screening EMRGs and Molecular Docking

**Figure 10:**
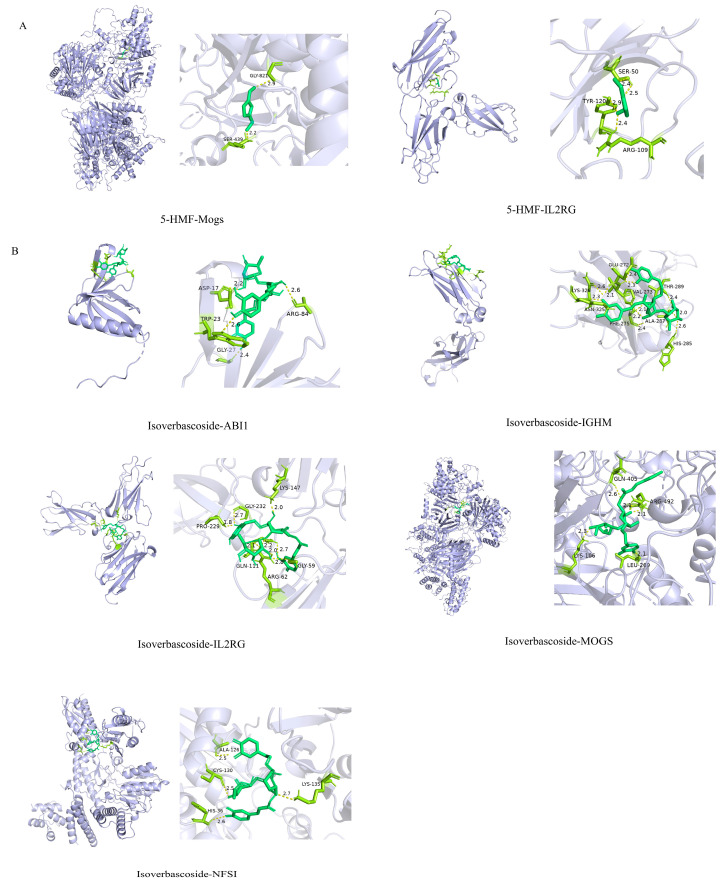
Molecular docking of 5-HMF and isoverbascoside. Note: (**A**): 5-HMF docking with gene molecules; (**B**): isoverbascoside docking with gene molecules.

Through LASSO regression analysis, three core EMRGs were screened in the 5-HMF treatment group, while four were identified in the isoverbascoside group. Molecular docking analysis predicted potential binding affinities between the compounds and key targets: 5-HMF showed docking scores of −5.6 (MOGS/5MHF), −3.5 (NFS1/8PK8), −3.9 (ABI1/7LXE), −4.4 (IGHM/3C2S), and −5.8 kcal/mol (IL2RG/7S2R), while isoverbascoside exhibited scores of −9.2 (MOGS), −6.6 (NFS1/8PK8), −7.3 (ABI1/7LXE), −7.3 (IGHM), and −8.5 kcal/mol (IL2RG/7S2R). According to computational criteria, scores ≤ −5.0 kcal/mol suggest spontaneous binding potential. Isoverbascoside may bind MOGS/5MHF, NFS1/8PK8, ABI1/7LXE, IGHM/3C2S, and IL2RG/7S2R with high affinity (all scores < −5.0 kcal/mol), 5-HMF shows moderate-to-high affinity for MOGS/5MHF and IL2RG/7S2R (scores ≤ −5.0 kcal/mol) (Figure 10). Subsequently, molecular docking simulations were performed to assess the binding potential of 5-HMF and verbascoside to key proteins involved in autophagy and apoptosis (BCL-2, MTOR, and BECN1). According to conventional scoring thresholds, verbascoside showed strong binding potential (all scores < −5.0 kcal/mol) for all three targets, indicating high-affinity interactions. In contrast, the docking results for 5-HMF did not suggest comparably stable binding under the same computational criteria. Full structural and energetic details of these docking simulations are provided in Supplementary Figure S1 for specific information. It should be explicitly stated that these in silico docking results are hypothesis-generating only and require further experimental validation.

### 3.12. Effects of 5-HMF and Isoverbascoside on H_2_O_2_ Induced Pancreatic β Cell Apoptosis

**Figure 11:**
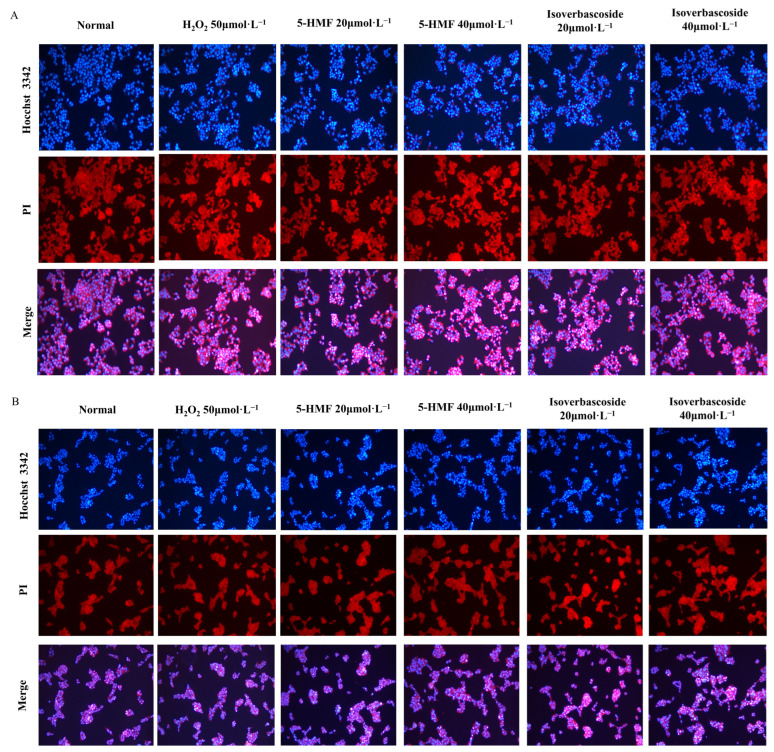
The effects of 5-HMF and Isoverbascoside on apoptosis of INS-1 and MIN6 cells induced by H_2_O_2_. Note: (**A**): Hoechst 33342 staining image of INS-1 cells; (**B**): Hoechst 33342 staining pattern of MIN6 cells.

The Hoechst 33342 fluorescent dye has the ability to permeate the cell membrane and intercalate with double-stranded DNA, thereby giving off blue fluorescence. The normal staining morphology of the cell nucleus is circular, with complete cell morphology and structure, uniform light blue fluorescence, and deep blue particles inside. In the H_2_O_2_ model group, a reduction in cell volume, loss of intercellular connections, marked nuclear shrinkage, intense blue fluorescence staining, and the presence of dense plaques or fragments were witnessed, indicating significant cell apoptosis (Figure 11).

**Figure 12:**
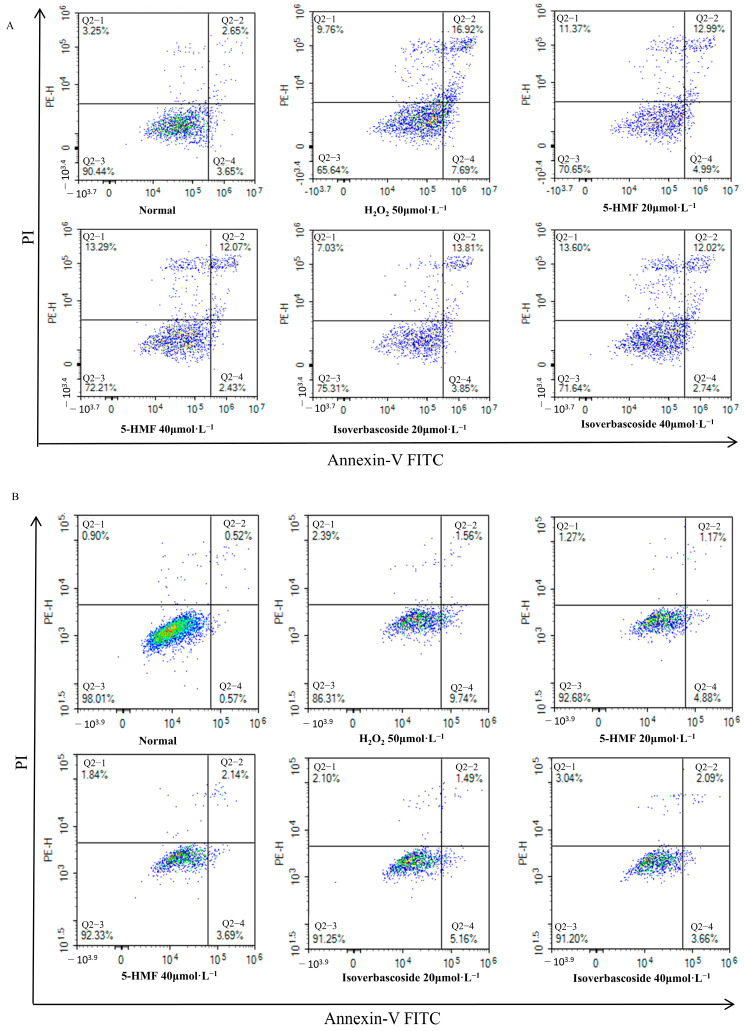
The effects of 5-HMF and isoverbascoside on H_2_O_2_-induced apoptosis of INS-1 and MIN6 cells. (**A**): INS-1 cell flow cytometry; (**B**): MIN6 cell flow cytometry.

Flow cytometry analyses disclosed that the normal apoptosis rate (Q2 + Q4) of INS-1 cells was 6.30%, while the apoptosis rate (Q2 + Q4) in the model group was 24.61%. For MIN6 cells, the normal apoptosis rate (Q2 + Q4) was 1.09%, in contrast to an apoptosis rate (Q2 + Q4) of 11.3% in the model group. Compared with the H_2_O_2_ group, 5-HMF significantly reduced apoptosis in both INS-1 and MIN6 cells at both 20 μmol·L^−1^ (*p* < 0.01) and 40 μmol·L^−1^ (*p* < 0.01), as did isoverbascoside at 20 μmol·L^−1^ (*p* < 0.01) and 40 μmol·L^−1^ (*p* < 0.01), indicating that both 5-HMF and isoverbascoside can effectively protect INS-1 and MIN6 cells, alleviate oxidative damage-induced apoptosis, and the relief effect shows a certain dose dependence, with high doses having stronger relief effects than low doses (Figure 12).

### 3.13. 5-HMF and Isoverbascoside Effects on Cellular Autophagy and Apoptosis

The immunofluorescence technique was employed to determine the localization and relative fluorescence intensity of the LC3B protein in INS-1 and MIN6 cells. The findings indicated that, in comparison to the control group, treatment with 5-HMF at a concentration of 40 μmol·L^−1^ and isoverbascoside at the same concentration resulted in an enhancement of LC3B protein expression, as evidenced by increased red fluorescence intensity in the cytoplasm of INS-1 cells. Similarly, the relative fluorescence intensity of LC3B protein in MIN6 cells exhibited an upward trend following treatment with 5-HMF and isoverbascoside. These results suggest that 5-HMF and isoverbascoside effectively augment the expression of LC3B protein in both INS-1 and MIN6 cells.

**Figure 13:**
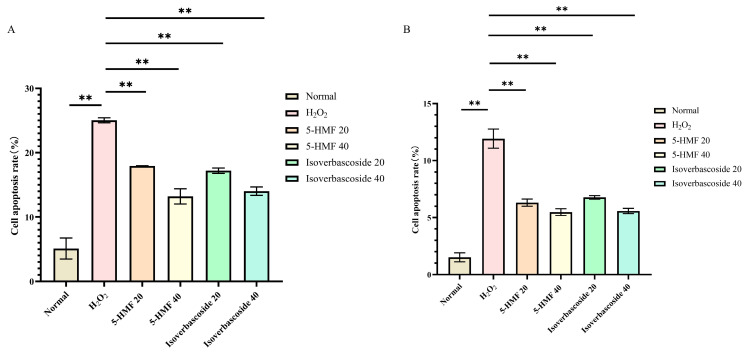
The effects of 5-HMF and isoverbascoside on H_2_O_2_-induced apoptosis of INS-1 and MIN6 cells. (**A**): INS-1 cell flow cytometry; (**B**): MIN6 cell flow cytometry ($\bar{x}\pm s$, *n* = 3, ** *p* < 0.01).

Analysis of autophagy-related protein expression in INS-1 cells revealed that both 5-HMF at 20 μmol·L^−1^ (*p* < 0.0001) and 40 μmol·L^−1^ (*p* < 0.0001), as well as isoverbascoside at 20 μmol·L^−1^ (*p* < 0.0001) and 40 μmol·L^−1^ (*p* < 0.0001), significantly promoted Beclin1 protein expression compared with the H_2_O_2_ group. This trend was also observed in MIN6 cells, where the expression of various proteins showed the same trend as in INS-1 cells (Figure 13).

**Figure 14:**
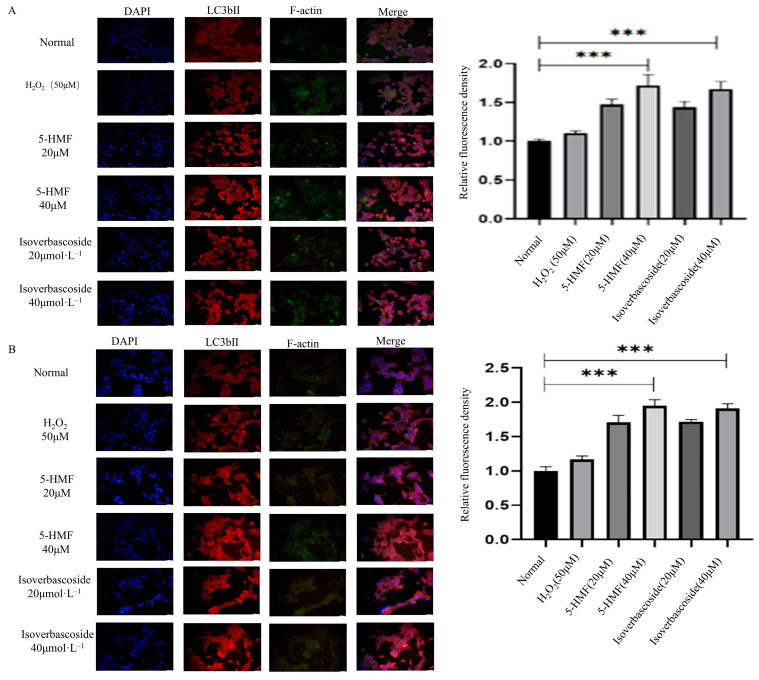

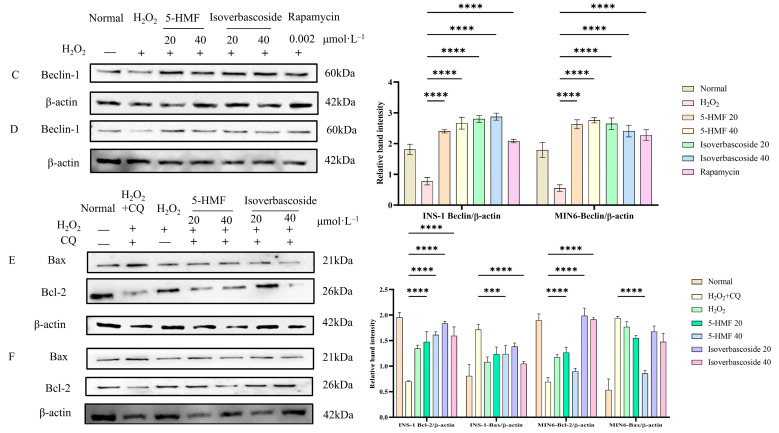
The effects of 5-HMF and Isoverbascoside in INS-1 and MIN6 cells (*n* = 3, *** *p* < 0.001, p **** < 0.0001). Note: (**A**) immunofluorescence staining of INS-1 cells; (**B**) immunofluorescence staining of MIN6 cells; (**C**) the effects of 5-HMF and isoverbascoside on autophagy-related protein expression in INS-1 cells; (**D**) the effects of 5-HMF and isoverbascoside on autophagy-related protein expression in MIN6 cells; (**E**) the effects of 5-HMF and isoverbascoside on apoptosis-related protein expression intensity in INS-1 cells; (**F**) the effects of 5-HMF and isoverbascoside on apoptosis-related protein expression intensity in MIN6 cells.

Compared with the H_2_O_2_ + CQ group in INS-1 cells, apoptosis-related protein analysis showed 5-HMF 40 μmol·L^−1^ (*p* < 0.05) and isoverbascoside 40 μmol·L^−1^ (*p* < 0.01) significantly downregulated Bax expression, while 5-HMF at 20 μmol·L^−1^ (*p* < 0.001) and 40 μmol·L^−1^ (*p* < 0.01) and isoverbascoside at 20 μmol·L^−1^ (*p* < 0.0001) and 40 μmol·L^−1^ (*p* < 0.0001) upregulated Bcl-2 expression. This trend was also observed in MIN6 cells, where the expression of various proteins showed the same trend as in INS-1 cells (Figure 14).

## 4. Discussion

In the pathological process of insulin resistance in type 2 diabetes (T2DM), autophagy dysfunction and increased apoptosis are common phenomena [35]. Dysautophagy can lead to the accumulation of intracellular metabolic waste, increased oxidative stress, and disrupted insulin signaling, thereby exacerbating insulin resistance [36,37]. Excessive apoptosis of pancreatic beta cells directly weakens insulin secretion ability and jointly promotes the progression of T2DM [38,39]. Therefore, maintaining a dynamic balance between autophagy and apoptosis has become an important target for regulating pancreatic beta cell function and glucose metabolism homeostasis.

Prepared Rehmannia glutinosa, as a commonly used traditional Chinese medicine with nourishing yin and kidney functions, has been proven to have antioxidant, anti-inflammatory, and insulin sensitivity regulating effects with its active ingredient 5-HMF and isoverbascoside [40,41]. This study takes this as a starting point to further explore whether these two components play a protective role in T2DM-related cell models by regulating autophagy–apoptosis balance [42,43]. This study takes this as a starting point to further explore whether these two components play a protective role in T2DM-related cell models by regulating autophagy–apoptosis balance.

In this experiment, H_2_O_2_ was utilized to induce oxidative damage in pancreatic β cells, followed by separate treatments with 5-HMF and isoverbascoside. Through the integration of proteomics and transcriptomics, the current study aims to clarify the effects of 5-HMF and isoverbascoside on the differential expression of genes and proteins in oxidative-damaged cells. GO enrichment analysis suggested that the identified differential factors are extensively involved in various biological functions, including cell signal transduction, cellular metabolism, enzymatic activity, and nitrogen metabolism. Additionally, KEGG enrichment analysis revealed that these differential factors are mainly concentrated in crucial pathways such as insulin secretion and the MAPK signaling pathway. The aforementioned findings imply that 5-HMF and isoverbascoside have direct impacts on cellular health and metabolic functions, thereby influencing T2DM by modulating insulin signaling, insulin functionality, endoplasmic reticulum stress, oxidative damage, autophagy, and apoptosis. Notably, molecular docking provided a structural hypothesis for these differential effects: isoverbascoside showed high in silico affinity for multiple targets (MOGS, NFS1, ABI1, IGHM, IL2RG), suggesting a potential multi-target mechanism, whereas 5-HMF displayed more selective binding, primarily to MOGS and IL2RG. This computational evidence aligns with the observed phenotypic differences and prioritizes MOGS as a candidate for future experimental validation. At the functional level, both compounds appear to attenuate apoptosis, as evidenced by the modulation of Bax/Bcl-2 protein ratios. Crucially, the use of the autophagy inhibitor chloroquine (CQ) reversed this anti-apoptotic effect, substantiating that the protection is mediated, at least in part, through the activation of autophagy. This interplay suggests that 5-HMF and isoverbascoside help restore cellular homeostasis under stress by promoting pro-survival autophagy, thereby suppressing deleterious apoptosis—a mechanism of high therapeutic relevance for preserving β-cell mass in T2DM. Previous studies, including preliminary findings from our group, have demonstrated that RRP alleviates insulin resistance in the liver, skeletal muscle, and adipose tissue, while modulating energy metabolism across multiple organs. However, the precise molecular mechanisms remain to be fully understood. This study provides additional evidence that RRP-derived monomers contribute to β-cell preservation through modulation of the autophagy–apoptosis pathway Beclin-1/Bcl-2-Bax, offering significant clinical insights. These findings provide a molecular basis for the use of RRP in traditional formulations such as Liuwei Dihuang decoction, suggesting that targeted monomer enrichment could optimize herbal prescriptions and potentially delay the onset of insulin dependence.

The current study has several limitations that warrant consideration. While the INS-1 and MIN6 cell lines are valuable for mechanistic exploration, they lack the cellular heterogeneity and physiological microenvironment of native pancreatic islets, which may constrain direct translation to in vivo systems. Furthermore, the acute H_2_O_2_-induced oxidative stress model, though reproducible, may not fully recapitulate the chronic, multifactorial metabolic dysregulation characteristic of human T2DM progression. Regarding experimental design, the study focused on comparing protective effects under oxidative stress and did not include a separate drug-only control group (treated with the highest protective dose without H_2_O_2_), limiting our ability to discern whether the compounds modulate basal autophagy or viability independently of stress. Regarding autophagy assessment, our conclusion that autophagy contributes to the cytoprotective effect remains inferential, as we did not provide direct evidence of autophagic flux—such as quantifying the LC3II/I ratio or p62 degradation—or employ genetic validation. Future studies should include flux assays and gain/loss-of-function experiments to establish causality. For apoptosis, although the data are included in the corresponding figure, the primary Western blot results demonstrating the drug-mediated modulation of Bax/Bcl-2 in the absence of the CQ inhibitor were not detailed in the text. Additionally, the depth of mechanistic interpretation is constrained by incomplete integration between the transcriptomic and proteomic datasets. Therefore, future investigations should prioritize validating the therapeutic potential of 5-HMF and isoverbascoside in well-characterized T2DM animal models while systematically defining their pharmacokinetic and safety profiles. Concurrently, in vitro studies would benefit from incorporating the aforementioned control groups and extended molecular markers to more fully dissect the stress-specificity and detailed mechanisms of action. These essential preclinical steps are crucial for evaluating the translational relevance of these compounds in T2DM management.

## 5. Conclusions

5-HMF and isoverbascoside (derived from RRP) regulate the expression balance of autophagy and apoptosis-related proteins in INS-1 and MIN6 cells, activating autophagy and inhibiting apoptosis through the Beclin-1/BCl-2 pathway, thereby protecting pancreatic β cells and achieving therapeutic effects on T2DM.

## Figures and Tables

**Table 1:** Statistical Table of Differential Genes and Proteins between INS-1 Cells and MIN6 Cells.

Difference Comparison Group			Normal vs. Control	5-HMF vs. Control	Isoverbascoside vs. Control	5-HMF vs. Isoverbascoside
Ins-1 cells	differential gene	UP Regulation	3474	195	323	108
		Down Regulation	2376	82	162	75
	differential protein	UP Regulation	151	5	6	8
		Down Regulation	27	18	9	7
Min6 cells	differential gene	UP Regulation	2696	134	85	93
		Down Regulation	2324	128	126	86
	differential protein	UP Regulation	250	8	25	26
		Down Regulation	194	6	8	13

## Data Availability

The raw data supporting the conclusions of this article will be made available by the authors on request. The transcriptome data generated have been deposited to National Center for Biotechnology Information (NCBI) under the BioProject number PRJNA1310232. The mass spectrometry proteomics data have been deposited to the ProteomeXchange Consortium (https://proteomecentral.proteomexchange.org, accessed on 26 August 2025) via the iProX partner repository with the dataset identifier PXD067713. The other data generated in the present study may be requested from the corresponding author.

## Supplementary Materials

The following supporting information can be downloaded at: https://www.mdpi.com/article/10.3390/metabo16010048/s1, Supplementary Table S1: GO functional analysis of 5-HMF vs Control in transcriptomics of INS-1 cells; Supplementary Table S2: GO functional analysis of Isoverbascoside vs Control in transcriptomics of INS-1 cells; Supplementary Table S3: GO functional analysis of 5-HMF vs. Control in transcriptomics of Min6 cell; Supplementary Table S4: GO functional analysis of Isoverbascoside vs. Control in transcriptomics of Min6 cell; Supplementary Table S5: KEGG Pathway Analysis of 5-HMF vs. Control Group in Proteomics of INS-1 Cell; Supplementary Table S6: KEGG Pathway Analysis of Isoverbascoside vs Control Group in Proteomics of INS-1 Cell; Supplementary Table S7: KEGG Pathway Analysis of 5-HMF vs. Control Group in Proteomics of Min6 cell; Supplementary Table S8: KEGG Pathway Analysis of Isoverbascoside vs. Control Group in Proteomics of Min6 cell; Supplementary Figure S1: Docking results of autophagy apoptosis related protein molecules.

## Abbreviations

The following abbreviations are used in this manuscript:

INS-1	Islet cell tumor cells
H_2_O_2_	Hydrogen peroxide
MIN6	Mouse pancreatic beta
TCM	Traditional Chinese Medicine
DM	Diabetes mellitus
STZ	Streptozotocin
FBG	Fasting blood glucose
OGTT	Oral Glucose Tolerance Test
AUC	Area Under the Curve
ITT	Insulin Tolerance Test
NC	Nitrocellulose
PCoA	Principal coordinate analysis
LEfSe	Linear discriminant analysis effect size
SEM	Standard Error of the Mean
ANOVA	Analysis of variance
BPC	Base Peak Chromatogram
OTU	Operational taxonomic unit
IR	Insulin Resistance
SCFAs	Short-chain fatty acids
5-HMF	5-Hydroxymethylfurfural
WB	Western blot
UPLC-HRMS	Ultra-high-performance liquid chromatography high-resolution mass spectrometry
GO	Gene Ontology
KEGG	Kyoto Encyclopedia of Genes and Genomes
CTD	Comparative Toxicogenomics Database
PICRUSt	Phylogenetic Investigation of Communities by Reconstruction of Unobserved States
AMPK	adenosine 5′-monophosphateactivated protein kinase
P-AMPK	phosphated adenosine 5′-monophosphateactivated protein kinase
ROS	Reactive oxygen species
CQ	chloroquine
FBS	Fetal Bovine Serum
ABI1	Abelson Interactor 1
IGHM	Immunoglobulin Heavy Constant Mu
mTOR	Mechanistic Target of Rapamycin
IL2RG	Interleukin-2 Receptor Subunit Gamma
BCL-2	receptors B-cell lymphoma 2
