# Supplementary material for: 5-Hydroxymethylfurfural and Isoverbascoside Alleviate Oxidative Damage INS-1 and MIN6 β-Cells by Activating Autophagy and Inhibiting Apoptosis
Source: Metabolites. 2026 Jan 6;16(1):48. doi: 10.3390/metabo16010048 (PMC12843818; doi:10.3390/metabo16010048)
Supplement: Supplementary file 1 [file metabolites-16-00048-s001.zip › supplementary File S1.pdf]

Supplementary Table S1 GO functional analysis of 5-HMF vs Control in transcriptomics of INS-1 cells

Term	Proteome_ids	Proteome_pvalue	Proteome_FDR	Transcriptome_ids
positive regulation of hydrogen peroxide-mediated programmed cell death	ENSRNOP00000021531(Endog)(Endog)	0.0143944657557096	0.31351372813085	ENSRNOG00000009371(Abl1)
positive regulation of hydrogen peroxide-induced cell death	ENSRNOP00000021531(Endog)	0.0215156480089799	0.31351372813085	ENSRNOG00000009371(Abl1)
Cdc42 protein signal transduction	ENSRNOP00000074944(Shtn1)	0.0215156480089799	0.31351372813085	ENSRNOG00000009371(Abl1)
syntaxin binding	ENSRNOP00000018717(Vps18),ENSRNOP00000068710(Sptan1)	0.0220282592974862	0.317206933883801	ENSRNOG00000009371(Abl1)
positive regulation of response to reactive oxygen species	ENSRNOP00000021531(Endog)	0.0285865948058868	0.32700927142435	ENSRNOG00000009371(Abl1)
regulation of hydrogen peroxide-mediated	ENSRNOP00000021531(Endog)	0.0285865948058868	0.32700927142435	ENSRNOG00000009371(Abl1)

programmed cell death				
programmed cell death in response to reactive oxygen species	ENSRNOP00000021531(Endog)	0.0356076519420341	0.34590290457976	ENSRNOG00000009371(Abl1)
epithelial structure maintenance	ENSRNOP00000041873(Cxadr)	0.0356076519420341	0.34590290457976	ENSRNOG00000015518(Rbp4)
hydrogen peroxide-mediated programmed cell death	ENSRNOP00000021531(Endog)	0.0356076519420341	0.34590290457976	ENSRNOG00000009371(Abl1)
SNARE binding	ENSRNOP00000018717(Vps18),ENSRNOP00000068710(Sptan1)	0.0381571990380767	0.352141184997468	ENSRNOG00000009371(Abl1)
positive regulation of oxidative stress-induced cell death	ENSRNOP00000021531(Endog)	0.0425791628918507	0.352141184997468	ENSRNOG00000009371(Abl1)
positive regulation of response to oxidative stress	ENSRNOP00000021531(Endog)	0.0495014688237744	0.358519513847928	ENSRNOG00000009371(Abl1)

Supplementary Table S2 GO functional analysis of Isoverbascoside vs Control in transcriptomics of INS-1 cells

Term	Proteome_ids	Proteome_pvalue	Proteome_FDR	Transcriptome_ids
response to salt stress	ENSRNOP00000024917(Agt)	0.0258907998655953	0.207993554901122	ENSRNOG00000058646(Zfp36l1)

Supplementary Table S3 GO functional analysis of 5-HMF vs Control in transcriptomics of Min6 cell

Term	Proteome_ids	Proteome_pvalue	Proteome_FDR	Transcriptome_ids
positive regulation of mRNA metabolic process	Q9CQ49(Ncbp2),Q9CQ49(Qki)	0.0335524315773982	0.288615655292915	ENSMUSG00000042487(Leo1),ENSMUSG00000061286(Exosc5),ENSMUSG00000027714(Exosc9),ENSMUSG00000037070(Rbmxl1),ENSMUSG00000022120(Obi1),ENSMUSG00000021039(Snw1),ENSMUSG00000015656(Hspa8),ENSMUSG00000026234(Ncl),ENSMUSG00000031167(Rbm3),ENSMUSG00000002227(Mov10),ENSMUSG00000041459(Tardbp),ENSMUSG00000034610(Tut4)
cellular nitrogen compound catabolic process	Q9CQ49(Ncbp2),Q91WG5(Prkag2i),P15208(Insr),Q9CQ49(Qki)	0.0352339538394 5	0.288615655292915	ENSMUSG00000024360(Etf1),ENSMUSG00000030655(Smg1),ENSMUSG00000030929(Eri2),ENSMUSG00000027714(Exosc9),ENSMUS

				G00000020719(Ddx5),ENSMUSG00000024120(Lrpprc),ENSMUSG00000027259(Adal),ENSMUSG00000021238(Aldh6a1),ENSMUSG00000042462(Dctpp1),ENSMUSG00000015337(Endog),ENSMUSG00000061286(Exosc5),ENSMUSG00000033991(Ttc37),ENSMUSG00000016018(Mtrex),ENSMUSG00000040482(Dxo),ENSMUSG00000091625(Lsm5),ENSMUSG00000020256(Aldh1l2),ENSMUSG00000022571(Pycrl),ENSMUSG00000034610(Tut4),ENSMUSG00000042396(Rbm7),ENSMUSG00000021595(Nsun2),ENSMUSG00000069806(Cacng7),ENSMUSG00000025962(Fastkd2),ENSMUSG00000028959(Fastk),ENSMUSG00000032047(Acat1),ENSMUSG00000020680(Taf15),ENSMUSG00000002227(Mov10),ENSMUSG00000041459(Tardbp),ENSMUSG00000042699(Dhx9),ENSMUSG00000068823(Csde1),ENSMUSG00000034218(Atm),ENSMUSG00000039449(Prpf18),ENSMUSG00000031403(Dkc1),ENSMUSG00000027201(Myef2)
heterocycle catabolic process	Q9CQ49(Ncbp2),Q91WG5(Prkag2i)(Prkag2i),P15208(Insr),Q9CQ49(Qki)	0.03523395383945	0.288615655292915	ENSMUSG00000024360(Etf1),ENSMUSG00000030655(Smg1),ENSMUSG00000030929(Eri2),ENSMUSG00000027714(Exosc9),ENSMUSG00000020719(Ddx5),ENSMUSG00000024120(Lrpprc),ENSMUSG00000027259(Adal),ENS

				MUSG00000021238(Aldh6a1),ENSMUSG00000042462(Dctpp1),ENSMUSG00000015337(Endog),ENSMUSG00000061286(Exosc5),ENSMUSG00000033991(Ttc37),ENSMUSG00000016018(Mtrex),ENSMUSG00000040482(Dxo),ENSMUSG00000091625(Lsm5),ENSMUSG00000020256(Aldh1l2),ENSMUSG00000022571(Pycrl),ENSMUSG00000034610(Tut4),ENSMUSG00000042396(Rbm7),ENSMUSG00000021595(Nsun2),ENSMUSG00000069806(Cacng7),ENSMUSG00000025962(Fastkd2),ENSMUSG00000028959(Fastk),ENSMUSG00000032047(Acat1),ENSMUSG00000020680(Taf15),ENSMUSG00000002227(Mov10),ENSMUSG00000041459(Tardbp),ENSMUSG00000042699(Dhx9),ENSMUSG00000068823(Csde1),ENSMUSG00000034218(Atm),ENSMUSG00000039449(Prpf18),ENSMUSG00000031403(Dkc1),ENSMUSG00000027201(Myef2)
translesion synthesis	Q9CQX4(Pclaf)	0.036334482236746	0.288615655292915	ENSMUSG00000021668(Polk),ENSMUSG00000026082(Rev1),ENSMUSG00000037474(Dtl),ENSMUSG00000063268(Parp10),ENSMUSG00000027342(Pcna)
regulation of cellular catabolic process	Q91WG5(Prkag2i),Q9DBL9,P15208(Insr),Q9CQ49(Qki),Q5XJY	0.0371437511839383	0.289943575262755	ENSMUSG00000024120(Lrpprc),ENSMUSG00000038916(Soga3),ENSMUSG00000025437(Usp33),ENSMUSG00000034218(Atm),ENSM

4(Parl)	USG00000070934(Rraga),ENSMUSG00000024958(Gpr137),ENSMUSG00000036273(Lrrk2),ENSMUSG00000028329(Xpa),ENSMUSG00000020572(Nampt),ENSMUSG00000023990(Tfeb),ENSMUSG00000040359(Ufl1),ENSMUSG00000026663(Atf6),ENSMUSG00000057177(Gsk3a),ENSMUSG00000038332(Sesn1),ENSMUSG00000024283(Wac),ENSMUSG00000000001(Gnai3),ENSMUSG00000036622(Atp13a2),ENSMUSG00000030655(Smg1),ENSMUSG00000024392(Bag6),ENSMUSG00000072872(Rybp),ENSMUSG00000031337(Mtm1),ENSMUSG00000028249(Sdcbp),ENSMUSG00000020184(Mdm2),ENSMUSG00000034636(Zyg11b),ENSMUSG00000022280(Rnf19a),ENSMUSG00000040782(Cop1),ENSMUSG00000031314(Taf1),ENSMUSG00000020738(Sumo2),ENSMUSG00000021595(Nsun2),ENSMUSG00000069806(Cacng7),ENSMUSG00000061286(Exosc5),ENSMUSG00000027714(Exosc9),ENSMUSG00000025962(Fastkd2),ENSMUSG00000028959(Fastk),ENSMUSG00000020680(Taf15),ENSMUSG00000032733(Snx33),ENSMUSG00000002227(Mov10),ENSMUSG00000026914(Psmd14),ENSMUSG00000041459(Tardbp),ENSMUSG00000053205(Styx),ENSMUSG00000042

				699(Dhx9),ENSMUSG00000031403(Dkc1),ENSMUSG00000046879(Irgm1),ENSMUSG00000021936(Mapk8),ENSMUSG00000032867(Fbxw8),ENSMUSG00000031633(Slc25a4),ENSMUSG00000015337(Endog),ENSMUSG00000021270(Hsp90aa1),ENSMUSG00000032050(Rdx),ENSMUSG00000024208(Uqcc2),ENSMUSG00000039753(Fbxl5),ENSMUSG00000021189(Atxn3),ENSMUSG00000021036(Sptlc2),ENSMUSG00000039958(Etfbkmt),ENSMUSG00000034610(Tut4),ENSMUSG00000026078(Pdcl3),ENSMUSG00000037075(Rnf139),ENSMUSG00000027201(Myef2)
cellular response to DNA damage stimulus	P18608(Hmgn1),Q9CQX4(Pclaf),Q9QZ67(Ppm1d),Q9D6J3(Yju2),Q9EP82(Wdr4)	0.0402993642383548	0.289943575262755	ENSMUSG00000021635(Rad17),ENSMUSG00000034218(Atm),ENSMUSG00000032409(Atr),ENSMUSG00000032534(Cep63),ENSMUSG00000020413(Hus1),ENSMUSG00000028329(Xpa),ENSMUSG00000041974(Spidr),ENSMUSG00000049932(H2ax),ENSMUSG00000051235(Gen1),ENSMUSG00000017146(Brca1),ENSMUSG00000032512(Wdr48),ENSMUSG00000027353(Mcm8),ENSMUSG00000026914(Psmd14),ENSMUSG00000068264(Ap5s1),ENSMUSG00000029283(Cdc7),ENSMUSG00000006678(Pola1),ENSMUSG00000037572(Wdhd1),ENSMUSG00000024151(Msh2),ENSMUSG0

0000021639(Gtf2h2),ENSMUSG00000045098(
Kmt5b),ENSMUSG00000040850(Psme4),ENS
MUSG00000025878(Uimc1),ENSMUSG00000
031422(Morf4l2),ENSMUSG00000014850(Ms
h3),ENSMUSG00000024054(Smchd1),ENSM
USG00000035726(Supt16),ENSMUSG000000
21668(Polk),ENSMUSG00000039187(Fanci),E
NSMUSG00000022906(Parp9),ENSMUSG000
00034206(Polq),ENSMUSG00000023932(Cdc5
l),ENSMUSG00000063268(Parp10),ENSMUS
G00000028629(Exo5),ENSMUSG0000004086
5(Ino80d),ENSMUSG00000059851(Kmt5c),E
NSMUSG00000030322(Mbd4),ENSMUSG000
00020380(Rad50),ENSMUSG00000040359(Ufl
1),ENSMUSG00000026082(Rev1),ENSMUSG
00000030655(Smg1),ENSMUSG00000029191(
Rfc1),ENSMUSG00000026526(Fh1),ENSMUS
G00000027342(Pcna),ENSMUSG00000021911
(Parg),ENSMUSG00000006599(Gtf2h1),ENS
MUSG00000020184(Mdm2),ENSMUSG00000
020326(Ccng1),ENSMUSG00000047281(Sfn),
ENSMUSG00000057789(Bak1),ENSMUSG00
000032216(Nedd4),ENSMUSG00000037474(D
tl),ENSMUSG00000038000(Acd),ENSMUSG0
0000024174(Pot1b),ENSMUSG00000007570(F
ance),ENSMUSG00000028410(Dnaja1),ENSM

				USG00000031027(Stk33),ENSMUSG00000021039(Snw1),ENSMUSG00000024392(Bag6),ENSMUSG00000020719(Ddx5),ENSMUSG00000042699(Dhx9),ENSMUSG00000056216(Cebpg),ENSMUSG00000000552(Zfp385a),ENSMUSG00000020228(Helb),ENSMUSG00000020152(Actr2),ENSMUSG00000035365(Parpbp),ENSMUSG00000037108(Zcwpw1),ENSMUSG00000031628(Casp3),ENSMUSG00000002661(Alkbh7),ENSMUSG00000020914(Top2a),ENSMUSG00000025616(Usp16),ENSMUSG00000016018(Mtrex),ENSMUSG00000024283(Wac),ENSMUSG00000031314(Taf1),ENSMUSG00000020898(Ctc1),ENSMUSG00000025899(Alkbh8),ENSMUSG00000020546(Stxbp4)
response to UV	P18608(Hmgn1),Q9CQX4(Pclaf)	0.0417699375135183	0.289943575262755	ENSMUSG00000028329(Xpa),ENSMUSG00000024151(Msh2),ENSMUSG00000057789(Bak1),ENSMUSG00000032216(Nedd4),ENSMUSG00000032409(Atr),ENSMUSG00000021668(Polk),ENSMUSG00000031314(Taf1),ENSMUSG00000027342(Pcna),ENSMUSG00000062997(Rpl35),ENSMUSG00000020184(Mdm2),ENSMUSG00000031628(Casp3),ENSMUSG00000021936(Mapk8),ENSMUSG00000037474(Dtl),ENSMUSG00000026082(Rev1),ENSMUSG00000020413(Hus1)

Supplementary Table S4 GO functional analysis of Isoverbascoside vs Control in transcriptomics of Min6 cell

Term	Proteome_ids	Proteome_pvalue	Proteome_FDR	Transcriptome_ids
cell differentiation in hindbrain	O70305(Atxn2),P48437(Prox1)	0.00577955623809478	0.319195787964958	ENSMUSG00000024259(Slc25a46),ENSMUSG00000034848(Ttc21b),ENSMUSG00000021557(Agtpbp1),ENSMUSG00000001911(Nfix)
lytic vacuole membrane	O54965(O54965),Q61144(Psen2),Q75N73(Slc39a14),Q8C0M0(Wdr59),Q9D6Y4(Borcs8)	0.0085153858337559	0.319195787964958	ENSMUSG00000045374(Wdr81),ENSMUSG00000019578(Ubxn6),ENSMUSG00000030720(Cln3),ENSMUSG00000022066(Entpd4b),ENSMUSG00000038280(Ostm1),ENSMUSG00000029571(Tmem106b),ENSMUSG00000036291(Ap5m1),ENSMUSG00000074811(Hps6),ENSMUSG00000046879(Irgm1),ENSMUSG00000053897(Slc39a8),ENSMUSG00000024958(Gpr137),ENSMUSG00000031007(Atp6ap2),ENSMUSG00000033253(Szt2),ENSMUSG00000027900(Dram2),ENSMUSG00000036622(Atp13a2),ENSMUSG00000023990(Tfeb),ENSMUSG00000034247(Plekhm1),ENSMUSG00000035206(Sppl2b),ENSMUSG00000020859(Spag9)
lysosomal membrane	O54965(O54965),Q61144(Psen2),Q75N73(Slc39a14),Q8C0M0(W	0.0085153858337559	0.319195787964958	ENSMUSG00000036622(Atp13a2),ENSMUSG00000045374(Wdr81),ENSMUSG00000019578(Ubxn6),ENSMUSG00000030720(Cln3),ENSM

	dr59),Q9D6Y4(Borcs8)			USG00000022066(Entpd4b),ENSMUSG00000038280(Ostm1),ENSMUSG00000029571(Tmem106b),ENSMUSG00000036291(Ap5m1),ENSMUSG00000074811(Hps6),ENSMUSG00000046879(Irgm1),ENSMUSG00000053897(Slc39a8),ENSMUSG00000024958(Gpr137),ENSMUSG00000031007(Atp6ap2),ENSMUSG00000033253(Szt2),ENSMUSG00000027900(Dram2),ENSMUSG00000023990(Tfeb),ENSMUSG00000034247(Plekhm1),ENSMUSG00000035206(Sppl2b),ENSMUSG00000020859(Spag9)
behavior	Q61144(Psen2),P15209(Ntrk2),Q8BUN9(Slc24a2),O08550(Kmt2b),Q80Z24(Negr1),Q8BM13(Olfm2),P49117(Nr2c2)	0.0142073369947333	0.319195787964958	ENSMUSG00000026959(Grin1),ENSMUSG00000020283(Pex13),ENSMUSG00000000552(Zfp385a),ENSMUSG00000030720(Cln3),ENSMUSG00000015476(Prrt1),ENSMUSG00000072964(Bhlhb9),ENSMUSG00000020745(Pafah1b1),ENSMUSG00000013662(Atad1),ENSMUSG00000035898(Uba6),ENSMUSG00000024261(Syt4),ENSMUSG00000006373(Pgrmc1),ENSMUSG00000035722(Abca7),ENSMUSG00000040907(Atp1a3),ENSMUSG00000056501(Cebpb),ENSMUSG00000024083(Pja2),ENSMUSG00000013787(Ehmt2),ENSMUSG00000070570(Slc17a7),ENSMUSG00000020431(Adcy1),ENSMUSG00000027273(Snap25),ENSMUSG00000032172(Olfm2),ENSMUSG00000022076(Klhl1),ENSM

				USG00000052512(Nav2),ENSMUSG00000022111(Uchl3),ENSMUSG00000022307(Oxr1),ENSMUSG00000021557(Agtpbp1),ENSMUSG00000026915(Strbp),ENSMUSG00000020152(Actr2),ENSMUSG00000039233(Tbce),ENSMUSG00000025889(Snca),ENSMUSG00000042105(Inpp5f),ENSMUSG00000030265(Kras),ENSMUSG00000021109(Hif1a),ENSMUSG00000067629(Syngap1),ENSMUSG00000020436(Gabrg2),ENSMUSG00000025235(Bbs4),ENSMUSG00000020308(Tpgs1),ENSMUSG00000024259(Slc25a46),ENSMUSG00000032245(Cln6),ENSMUSG00000031592(Pcm1),ENSMUSG00000029071(Dvl1),ENSMUSG00000036273(Lrrk2),ENSMUSG00000026904(Slc4a10),ENSMUSG00000020889(Nr1d1),ENSMUSG00000051444(Bbs12),ENSMUSG00000045532(C1ql1),ENSMUSG00000026097(Ormdl1),ENSMUSG00000041329(Atp1b2),ENSMUSG00000031696(Vps35),ENSMUSG00000034848(Ttc21b)
protein serine/threonine/tyrosine kinase activity	O09110(Map2k3),O35492(Clk3)	0.0205163101543193	0.319195787964958	ENSMUSG00000036273(Lrrk2),ENSMUSG00000038379(Ttk),ENSMUSG00000002409(Dyrk1b),ENSMUSG00000021936(Mapk8),ENSMUSG00000020385(Clk4),ENSMUSG00000028458(Tesk1)

learning or memory	P15209(Ntrk2),Q8BUN9(Slc24a2),O08550(Kmt2b),Q61144(Psen2)	0.0268272333002008	0.328522507662617	ENSMUSG00000026959(Grin1),ENSMUSG00000013662(Atad1),ENSMUSG00000035898(Uba6),ENSMUSG00000024261(Syt4),ENSMUSG00000006373(Pgrmc1),ENSMUSG00000035722(Abca7),ENSMUSG00000040907(Atp1a3),ENSMUSG00000056501(Cebpb),ENSMUSG00000024083(Pja2),ENSMUSG00000013787(Ehmt2),ENSMUSG00000070570(Slc17a7),ENSMUSG00000020431(Adcy1),ENSMUSG00000027273(Snap25),ENSMUSG00000030720(Cln3),ENSMUSG00000020152(Actr2),ENSMUSG00000030265(Kras),ENSMUSG00000021109(Hif1a),ENSMUSG00000067629(Syngap1),ENSMUSG00000045532(C1ql1),ENSMUSG00000000552(Zfp385a),ENSMUSG00000015476(Prrt1),ENSMUSG00000072964(Bhlhb9),ENSMUSG00000020745(Pafah1b1)
vacuolar membrane	O54965(O54965),Q61144(Psen2),Q75N73(Slc39a14),Q8C0M0(Wdr59),Q9D6Y4(Borcs8)	0.0280411028296632	0.328522507662617	ENSMUSG00000001750(Tcirg1),ENSMUSG00000045374(Wdr81),ENSMUSG00000022066(Entpd4b),ENSMUSG00000027602(Map1lc3a),ENSMUSG00000046879(Irgm1),ENSMUSG00000031007(Atp6ap2),ENSMUSG00000036622(Atp13a2),ENSMUSG00000025907(Rb1cc1),ENSMUSG00000019578(Ubxn6),ENSMUSG00000030720(Cln3),ENSMUSG00000038280(Ostm1),ENSMUSG00000029571(Tmem106b),ENSMU

				SG00000036291(Ap5m1),ENSMUSG00000074811(Hps6),ENSMUSG00000053897(Slc39a8),ENSMUSG00000024958(Gpr137),ENSMUSG00000033253(Szt2),ENSMUSG00000027900(Dram2),ENSMUSG00000023990(Tfeb),ENSMUSG00000034247(Plekhm1),ENSMUSG00000035206(Sppl2b),ENSMUSG00000020859(Spag9),ENSMUSG00000070934(Rraga)
dendritic shaft	Q61144(Psen2),Q9Z0G0(Gipc1)	0.0344059737135736	0.328522507662617	ENSMUSG00000024873(Cnih2),ENSMUSG00000000881(Dlg3),ENSMUSG00000017314(Mpp2),ENSMUSG00000067629(Syngap1),ENSMUSG00000029763(Exoc4),ENSMUSG00000020331(Hcn2),ENSMUSG00000015222(Map2),ENSMUSG00000006932(Ctnnb1)
cognition	Q61144(Psen2),P15209(Ntrk2),Q8BUN9(Slc24a2),O08550(Kmt2b)	0.0388241356714299	0.355400392636069	ENSMUSG00000026959(Grin1),ENSMUSG00000000552(Zfp385a),ENSMUSG00000030720(Cln3),ENSMUSG00000015476(Prrt1),ENSMUSG00000072964(Bhlhb9),ENSMUSG00000020745(Pafah1b1),ENSMUSG00000013662(Atad1),ENSMUSG00000035898(Uba6),ENSMUSG00000024261(Syt4),ENSMUSG00000006373(Pgrmc1),ENSMUSG00000035722(Abca7),ENSMUSG00000040907(Atp1a3),ENSMUSG00000056501(Cebpb),ENSMUSG00000024083(Pja2),ENSMUSG00000013787(Ehmt2),ENSMUSG00000070570(Slc17a7),ENSMUSG00000020431(Adcy

				1),ENSMUSG00000027273(Snap25),ENSMUSG00000020152(Actr2),ENSMUSG00000030265(Kras),ENSMUSG00000021109(Hif1a),ENSMUSG00000067629(Syngap1),ENSMUSG00000045532(C1ql1),ENSMUSG00000031232(Magt1),ENSMUSG00000022141(Nipbl)
nuclear inner membrane	O54965(O54965),Q61144(Psen2)	0.0423885205504647	0.35746187237642	ENSMUSG00000038271(Iffo1),ENSMUSG00000022858(Tra2b),ENSMUSG00000024799(Tm7sf2),ENSMUSG00000024120(Lrpprc),ENSMUSG00000045205(Dpy19l4),ENSMUSG00000039886(Tmem120a),ENSMUSG00000026960(Arl6ip6)
optic nerve development	Q9D8Y1(Tmem126a)	0.0462707848268766	0.35746187237642	ENSMUSG00000024259(Slc25a46),ENSMUSG00000001750(Tcirg1),ENSMUSG00000052512(Nav2)

Supplementary Table S5 KEGG Pathway Analysis of 5-HMF vs Control Group in Proteomics of INS-1 Cell

**Map.Name**	**Proteome_ids**	**Proteome_pvalue**	**Proteome_FDR**	**Transcriptome_ids**
Autophagy - animal	ENSRNOP00000018717(Vps18),ENSRNOP00000032429(Map1lc3a)	0.0672844772009594	0.240894924353428	ENSRNOG00000034246(Rps27a)

Mitophagy - animal	ENSRNOP00000032429(Map1lc3a)	0.257873843285207	0.394395289730316	ENSRNOG00000034246(Rps27a)
Kaposi sarcoma-associated herpesvirus infection	ENSRNOP00000032429(Map1lc3a)	0.310162287998218	0.403210974397684	ENSRNOG00000034246(Rps27a)
Protein processing in endoplasmic reticulum	ENSRNOP00000076327(Dnajc3)	0.425648739593132	0.503039419519156	ENSRNOG00000008648(Mogs)
Pathways of neurodegeneration - multiple diseases	ENSRNOP00000032429(Map1lc3a)	0.753466856725272	0.753466856725272	ENSRNOG00000034246(Rps27a)

Supplementary Table S6 KEGG Pathway Analysis of Isoverbascoside vs Control Group in Proteomics of INS-1 Cell

**Map.Name**	**Proteome_ids**	**Proteome_pvalue**	**Proteome_FDR**	**Transcriptome_ids**
Endocytosis	ENSRNOP00000035138(Clta)|ENSRNOP00000073332(Arpc5)	0.0997259446526135	0.27970080094548	ENSRNOG00000003954(Il2rg)
Regulation of actin cytoskeleton	ENSRNOP00000073332(Arpc5)	0.303916191244379	0.362361612637528	ENSRNOG00000047931(Tmsb4x)
Pathways in cancer	ENSRNOP00000024917(Agt)	0.494045638932854	0.510513826897282	ENSRNOG00000003954(Il2rg)

Supplementary Table S7 KEGG Pathway Analysis of 5-HMF vs Control Group in Proteomics of Min6 cell

Map.Name	Proteome_ids	Proteome_pvalue	Proteome_FDR	Transcriptome_ids
Regulation of lipolysis in adipocytes	P15208(Insr)|Q9DBL9(Abhd5)	0.00307825347632346	0.0921481214377654	ENSMUSG00000019699(Akt3)|ENSMUSG00000000001(Gnai3)
Cell adhesion molecules	Q80Z24(Negr1)|Q8BIF0(Cd99l2)	0.00868594728094776	0.135817223437867	ENSMUSG00000033768(Nrxn2)|ENSMUSG00000073405(H2-T-ps)|ENSMUSG00000024448(H2-M10.1)|ENSMUSG00000025809(Itgb1)
Non-alcoholic fatty liver disease	P15208(Insr)|P17665(Cox7c)|Q91WG5(Prkag2)	0.0121019160360941	0.135817223437867	ENSMUSG00000019699(Akt3)|ENSMUSG00000004054(Map3k11)|ENSMUSG00000002603(Tgfb1)|ENSMUSG00000046709(Mapk10)|ENSMUSG00000031628(Casp3)|ENSMUSG00000083380(Ndufb4c)|ENSMUSG00000021936(Mapk8)|ENSMUSG00000026029(Casp8)|ENSMUSG00000030785(Cox6a2)|ENSMUSG00000024038(Ndufv3)|ENSMUSG00000041881(Ndufa7)|ENSMUSG00000057177(Gsk3a)|ENSMUSG00000025651(Uqcrc1)
Longevity regulating pathway - multiple species	P15208(Insr)|Q91WG5(Prkag2)	0.012812945607346	0.135817223437867	ENSMUSG00000019699(Akt3)|ENSMUSG00000030265(Kras)|ENSMUSG00000015656(Hspa8)|ENSMUSG00000011096(Akt1s1)

Longevity regulating pathway	P15208(Insr)|Q91WG5(Prkag2)	0.0212118340812203	0.187371201050779	ENSMUSG00000040760(Appl1)|ENSMUSG00000019699(Akt3)|ENSMUSG00000030265(Kras)|ENSMUSG00000038332(Sesn1)|ENSMUSG00000011096(Akt1s1)
FoxO signaling pathway	P15208(Insr)|Q91WG5(Prkag2)	0.0313370360682031	0.214896044268321	ENSMUSG00000020184(Mdm2)|ENSMUSG00000019699(Akt3)|ENSMUSG00000002603(Tgfb1)|ENSMUSG00000046709(Mapk10)|ENSMUSG00000034218(Atm)|ENSMUSG00000030265(Kras)|ENSMUSG00000021936(Mapk8)|ENSMUSG00000034793(G6pc3)
Insulin resistance	P15208(Insr)|Q91WG5(Prkag2)	0.0324371387574825	0.214896044268321	ENSMUSG00000019699(Akt3)|ENSMUSG00000046709(Mapk10)|ENSMUSG00000059316(Slc27a4)|ENSMUSG00000021936(Mapk8)|ENSMUSG00000034793(G6pc3)|ENSMUSG00000027932(Slc27a3)|ENSMUSG00000039515(Ptpa)
AMPK signaling pathway	P15208(Insr)|Q91WG5(Prkag2)	0.0417699375135183	0.245978520912941	ENSMUSG00000019699(Akt3)|ENSMUSG00000024777(Ppp2r5b)|ENSMUSG00000025202(Scd3)|ENSMUSG00000011096(Akt1s1)|ENSMUSG00000034793(G6pc3)
Ovarian steroidogenesis	P15208(Insr)	0.0464791491218201	0.246339490345646	ENSMUSG00000052914(Cyp2j6)
Ovarian steroidogenesis	P15208(Insr)	0.0464791491218201	0.246339490345646	ENSMUSG00000052914(Cyp2j6)
Circadian rhythm	Q91WG5(Prkag2)	0.0515126922374887	0.24745510024712	ENSMUSG00000020889(Nr1d1)|ENSMUSG00000055116(Arntl)

Insulin signaling pathway	P15208(Insr)|Q91WG5(Prkag2)	0.0560275698672724	0.24745510024712	ENSMUSG00000019699(Akt3)|ENSMUSG00000046709(Mapk10)|ENSMUSG00000030265(Kras)|ENSMUSG00000021936(Mapk8)|ENSMUSG00000034793(G6pc3)|ENSMUSG00000009406(Elk1)
Aldosterone-regulated sodium reabsorption	P15208(Insr)	0.0713912240781098	0.291056528933832	ENSMUSG00000030265(Kras)|ENSMUSG00000047281(Sfn)|ENSMUSG00000026576(Atp1b1)|ENSMUSG00000041329(Atp1b2)|ENSMUSG00000040907(Atp1a3)
Type II diabetes mellitus	P15208(Insr)	0.0860352121178761	0.325704731589102	ENSMUSG00000046709(Mapk10)|ENSMUSG00000021936(Mapk8)
p53 signaling pathway	Q9QZ67(Ppm1d)	0.100455847400556	0.354943994148632	ENSMUSG00000000552(Zfp385a)|ENSMUSG00000020184(Mdm2)|ENSMUSG00000034218(Atm)|ENSMUSG00000031628(Casp3)|ENSMUSG00000038332(Sesn1)|ENSMUSG00000047281(Sfn)|ENSMUSG00000020326(Ccng1)|ENSMUSG00000040782(Cop1)|ENSMUSG00000032409(Atr)|ENSMUSG00000026029(Casp8)
Hypertrophic cardiomyopathy	Q91WG5(Prkag2)	0.109947146448776	0.364199922611572	ENSMUSG00000002603(Tgfb1)|ENSMUSG00000020882(Cacnb1)|ENSMUSG00000029156(Sgcb)|ENSMUSG00000069806(Cacng7)|ENSMUSG00000025809(Itgb1)
Cytosolic DNA-sensing pathway	Q80UW8(Polr2e)	0.128640176434044	0.378773852833575	ENSMUSG00000031628(Casp3)|ENSMUSG00000026029(Casp8)
Pancreatic secretion	Q99P58(Rab27b)	0.128640176434044	0.378773852833575	ENSMUSG00000032839(Trpc1)|ENSMUSG00000026576(Atp1b1)|ENSMUSG00000028962(Slc4a2)|ENSMUSG00000041329(Atp1b2)|ENSMUSG000000

				40907(Atp1a3)
Diabetic cardiomyopathy	P15208(Insr)|P17665(Cox7c)	0.147241262755073	0.39951070669057	ENSMUSG00000064341(mt-Nd1)|ENSMUSG00000064356(mt-Atp8)|ENSMUSG00000064360(mt-Nd3)|ENSMUSG00000064368(mt-Nd6)|ENSMUSG00000019699(Akt3)|ENSMUSG00000002603(Tgfb1)|ENSMUSG00000031633(Slc25a4)|ENSMUSG00000046709(Mapk10)|ENSMUSG00000083380(Ndufb4c)|ENSMUSG00000031299(Pdha1)|ENSMUSG00000033220(Rac2)|ENSMUSG00000021936(Mapk8)|ENSMUSG00000030785(Cox6a2)|ENSMUSG00000024038(Ndufv3)|ENSMUSG00000039515(Ptpa)|ENSMUSG00000041881(Ndufa7)|ENSMUSG00000025651(Uqcrc1)|ENSMUSG00000038967(Pdk2)
Adipocytokine signaling pathway	Q91WG5(Prkag2)	0.164894369761445	0.39951070669057	ENSMUSG00000039656(Rxrb)|ENSMUSG00000019699(Akt3)|ENSMUSG00000046709(Mapk10)|ENSMUSG00000021936(Mapk8)|ENSMUSG00000034793(G6pc3)
Cardiac muscle contraction	P17665(Cox7c)	0.169322289327927	0.39951070669057	ENSMUSG00000020882(Cacnb1)|ENSMUSG00000069806(Cacng7)|ENSMUSG00000030785(Cox6a2)|ENSMUSG00000026576(Atp1b1)|ENSMUSG00000041329(Atp1b2)|ENSMUSG00000040907(Atp1a3)|ENSMUSG00000025651(Uqcrc1)
Thermogenesis	P17665(Cox7c)|Q91WG5(Prkag2)	0.171378702738138	0.39951070669057	ENSMUSG00000064341(mt-Nd1)|ENSMUSG00000064356(mt-Atp8)|ENSMUSG00000064360(mt-Nd3)|ENSMUSG00000064368(mt-Nd6)|ENSMUSG00

				000083380(Ndufb4c)|ENSMUSG00000030265(Kras)|ENSMUSG00000011096(Akt1s1)|ENSMUSG00000030785(Cox6a2)|ENSMUSG00000024038(Ndufv3)|ENSMUSG00000041881(Ndufa7)|ENSMUSG00000025651(Uqcrc1)|ENSMUSG00000020544(Cox11)
Nucleotide excision repair	Q80UW8(Polr2e)	0.186808096121035	0.39951070669057	ENSMUSG00000021639(Gtf2h2)|ENSMUSG00000029191(Rfc1)|ENSMUSG00000006599(Gtf2h1)|ENSMUSG00000028329(Xpa)|ENSMUSG00000027342(Pcna)
Leukocyte transendothelial migration	Q8BIF0(Cd99l2)	0.186808096121035	0.39951070669057	ENSMUSG00000033220(Rac2)|ENSMUSG00000025809(Itgb1)|ENSMUSG00000000001(Gnai3)|ENSMUSG00000006932(Ctnnb1)
Glycerophospholipid metabolism	Q8BSF4(Pisd)	0.195417036751017	0.39951070669057	ENSMUSG00000000301(Pemt)|ENSMUSG00000024978(Gpam)|ENSMUSG00000042632(Pla2g6)|ENSMUSG00000020910(Adprm)|ENSMUSG00000027346(Gpcpd1)
Calcium signaling pathway	Q99M08(Uncharacterized protein C4orf3 homolog)	0.203937844795435	0.39951070669057	ENSMUSG00000031633(Slc25a4)|ENSMUSG00000003752(Itpkc)|ENSMUSG00000038855(Itpkb)|ENSMUSG00000049686(Orai1)|ENSMUSG00000029231(Pdgfra)|ENSMUSG00000020178(Adora2a)|ENSMUSG00000023990(Tfeb)
Oxytocin signaling pathway	Q91WG5(Prkag2)	0.2207185405701	0.39951070669057	ENSMUSG00000020882(Cacnb1)|ENSMUSG00000030265(Kras)|ENSMUSG00000069806(Cacng7)|ENSMUSG00000009406(Elk1)|ENSMUSG00000000001(Gnai3)

Glucagon signaling pathway	Q91WG5(Prkag2)	0.224859983224288	0.39951070669057	ENSMUSG00000019699(Akt3)|ENSMUSG00000031299(Pdha1)|ENSMUSG00000034793(G6pc3)
Alcoholic liver disease	Q91WG5(Prkag2)	0.224859983224288	0.39951070669057	ENSMUSG00000019699(Akt3)|ENSMUSG00000046709(Mapk10)|ENSMUSG00000031628(Casp3)|ENSMUSG00000062908(Acadm)|ENSMUSG00000025202(Scd3)|ENSMUSG00000010025(Aldh3a2)|ENSMUSG00000021936(Mapk8)|ENSMUSG00000026029(Casp8)|ENSMUSG00000030281(Il17rc)|ENSMUSG00000006932(Ctnnb1)
Phospholipase D signaling pathway	P15208(Insr)	0.233079135439533	0.39951070669057	ENSMUSG00000019699(Akt3)|ENSMUSG00000030265(Kras)|ENSMUSG00000029231(Pdgfra)|ENSMUSG00000008859(Rala)|ENSMUSG00000026825(Dnm1)
Adherens junction	P15208(Insr)	0.237157055669383	0.39951070669057	ENSMUSG00000033060(Lmo7)|ENSMUSG00000044573(Acp1)|ENSMUSG00000033220(Rac2)|ENSMUSG00000006932(Ctnnb1)
HIF-1 signaling pathway	P15208(Insr)	0.241214011586759	0.39951070669057	ENSMUSG00000019699(Akt3)|ENSMUSG00000031299(Pdha1)
cGMP-PKG signaling pathway	P15208(Insr)	0.257234266233221	0.400982826775315	ENSMUSG00000019699(Akt3)|ENSMUSG00000031633(Slc25a4)|ENSMUSG00000026576(Atp1b1)|ENSMUSG00000000001(Gnai3)|ENSMUSG00000041329(Atp1b2)|ENSMUSG00000040907(Atp1a3)
Apelin signaling pathway	Q91WG5(Prkag2)	0.257234266233221	0.400982826775315	ENSMUSG00000019699(Akt3)|ENSMUSG00000030265(Kras)|ENSMUSG00000003923(Tfam)|ENSMUSG00000027602(Map1lc3a)|ENSMUSG00000008855(Hdac5)|ENSMUSG00000000001(Gnai3)

Huntington disease	P17665(Cox7c)|Q80UW8(Polr2e)	0.280521764926786	0.418404013991014	ENSMUSG00000064341(mt-Nd1)|ENSMUSG00000064356(mt-Atp8)|ENSMUSG00000064360(mt-Nd3)|ENSMUSG00000064368(mt-Nd6)|ENSMUSG00000032965(Ift57)|ENSMUSG00000031633(Slc25a4)|ENSMUSG00000046709(Mapk10)|ENSMUSG00000031628(Casp3)|ENSMUSG00000083380(Ndufb4c)|ENSMUSG00000040390(Map3k10)|ENSMUSG00000026229(Psmd1)|ENSMUSG00000003923(Tfam)|ENSMUSG00000040714(Klc3)|ENSMUSG00000021936(Mapk8)|ENSMUSG00000026029(Casp8)|ENSMUSG00000030785(Cox6a2)|ENSMUSG00000024038(Ndufv3)|ENSMUSG00000041881(Ndufa7)|ENSMUSG00000047126(Cltc)|ENSMUSG00000025651(Uqcrc1)|ENSMUSG00000026914(Psmd14)
Rap1 signaling pathway	P15208(Insr)	0.284486295035523	0.418404013991014	ENSMUSG00000019699(Akt3)|ENSMUSG00000030265(Kras)|ENSMUSG00000029231(Pdgfra)|ENSMUSG00000033220(Rac2)|ENSMUSG00000008859(Rala)|ENSMUSG00000027805(Pfn2)|ENSMUSG00000025809(Itgb1)|ENSMUSG00000030583(Sipa1l3)|ENSMUSG00000020178(Adora2a)|ENSMUSG00000000001(Gnai3)|ENSMUSG00000037533(Rapgef6)|ENSMUSG00000006932(Ctnnb1)
mRNA surveillance pathway	Q9CQ49(Ncbp2)	0.292093368257878	0.418404013991014	ENSMUSG00000020994(Pnn)|ENSMUSG00000024360(Etf1)|ENSMUSG00000041459(Tardbp)|ENSMUSG00000024777(Ppp2r5b)|ENSMUSG00000041781(Cpsf2)|ENSMUSG00000021111(Papola)|ENSM

				USG00000054256(Msi1)|ENSMUSG00000030655(Smg1)
Alzheimer disease	P15208(Insr)|P17665(Cox7c)	0.308181832636885	0.429832556046182	ENSMUSG00000064341(mt-Nd1)|ENSMUSG00000064356(mt-Atp8)|ENSMUSG00000064360(mt-Nd3)|ENSMUSG00000064368(mt-Nd6)|ENSMUSG00000019699(Akt3)|ENSMUSG00000052593(Adam17)|ENSMUSG00000031633(Slc25a4)|ENSMUSG00000046709(Mapk10)|ENSMUSG00000031628(Casp3)|ENSMUSG00000083380(Ndufb4c)|ENSMUSG00000030265(Kras)|ENSMUSG00000026229(Psmd1)|ENSMUSG00000053897(Slc39a8)|ENSMUSG00000040714(Klc3)|ENSMUSG00000021936(Mapk8)|ENSMUSG00000026029(Casp8)|ENSMUSG00000036856(Wnt4)|ENSMUSG00000030785(Cox6a2)|ENSMUSG00000024038(Ndufv3)|ENSMUSG00000020135(Apc2)|ENSMUSG00000026663(Atf6)|ENSMUSG00000041881(Ndufa7)|ENSMUSG00000006932(Ctnnb1)|ENSMUSG00000025651(Uqcrc1)|ENSMUSG00000026914(Psmd14)
mTOR signaling pathway	P15208(Insr)	0.318107319107207	0.431113296815809	ENSMUSG00000052459(Atp6v1a)|ENSMUSG00000019699(Akt3)|ENSMUSG00000030265(Kras)|ENSMUSG00000011096(Akt1s1)|ENSMUSG00000024170(Telo2)|ENSMUSG00000070934(Rraga)|ENSMUSG00000036856(Wnt4)|ENSMUSG00000028278(Rragd)|ENSMUSG00000033793(Atp6v1h)

Ras signaling pathway	P15208(Insr)	0.325368525898724	0.431113296815809	ENSMUSG00000019699(Akt3)|ENSMUSG00000046709(Mapk10)|ENSMUSG00000030265(Kras)|ENSMUSG00000029231(Pdgfra)|ENSMUSG00000024976(Shoc2)|ENSMUSG00000033220(Rac2)|ENSMUSG00000008859(Rala)|ENSMUSG00000021936(Mapk8)|ENSMUSG00000042632(Pla2g6)|ENSMUSG00000009406(Elk1)
Lysosome	Q69ZN6(Gnptab)	0.339667370433366	0.437510568631488	ENSMUSG00000021686(Ap3b1)|ENSMUSG00000028164(Manba)|ENSMUSG00000021824(Ap3m1)|ENSMUSG00000033128(Gga1)|ENSMUSG00000001750(Tcirg1)|ENSMUSG00000033379(Atp6v0b)|ENSMUSG00000033793(Atp6v1h)|ENSMUSG00000047126(Cltc)
Tight junction	Q91WG5(Prkag2)	0.346706488349481	0.437510568631488	ENSMUSG00000020782(Llgl2)|ENSMUSG00000046709(Mapk10)|ENSMUSG00000032050(Rdx)|ENSMUSG00000044279(Crb3)|ENSMUSG00000026341(Actr3)|ENSMUSG00000021936(Mapk8)|ENSMUSG00000000881(Dlg3)|ENSMUSG00000025809(Itgb1)|ENSMUSG00000020361(Hspa4)|ENSMUSG00000037533(Rapgef6)|ENSMUSG00000032216(Nedd4)|ENSMUSG00000036718(Micall2)|ENSMUSG00000020152(Actr2)|ENSMUSG00000027342(Pcna)
Nucleocytoplasmic transport	Q9CQ49(Ncbp2)	0.367391357285096	0.452831207816514	ENSMUSG00000020994(Pnn)|ENSMUSG00000042590(Ipo11)|ENSMUSG00000066232(Ipo7)|ENSMUSG00000040034(Nup43)|ENSMUSG00000012535(Tnpo3)|ENSMUSG00000034826(Nup54)|ENSMUS

				G00000018362(Kpna2)|ENSMUSG00000006005(Tpr)|ENSMUSG00000020738(Sumo2)|ENSMUSG00000002718(Cse1l)
PI3K-Akt signaling pathway	P15208(Insr)	0.384143812641806	0.462718683409448	ENSMUSG00000021270(Hsp90aa1)|ENSMUSG00000020184(Mdm2)|ENSMUSG00000019699(Akt3)|ENSMUSG00000057672(Pkn1)|ENSMUSG00000024777(Ppp2r5b)|ENSMUSG00000039481(Nrtn)|ENSMUSG00000030265(Kras)|ENSMUSG00000029231(Pdgfra)|ENSMUSG00000017146(Brca1)|ENSMUSG00000034793(G6pc3)|ENSMUSG00000025809(Itgb1)|ENSMUSG00000028530(Jak1)|ENSMUSG00000068758(Il3ra)|ENSMUSG00000026193(Fn1)
MAPK signaling pathway	P15208(Insr)	0.393988572927992	0.464030985892969	ENSMUSG00000019699(Akt3)|ENSMUSG00000004054(Map3k11)|ENSMUSG00000002603(Tgfb1)|ENSMUSG00000020882(Cacnb1)|ENSMUSG00000046709(Mapk10)|ENSMUSG00000031628(Casp3)|ENSMUSG00000039481(Nrtn)|ENSMUSG00000030265(Kras)|ENSMUSG00000040390(Map3k10)|ENSMUSG00000015656(Hspa8)|ENSMUSG00000029231(Pdgfra)|ENSMUSG00000069806(Cacng7)|ENSMUSG00000033220(Rac2)|ENSMUSG00000021936(Mapk8)|ENSMUSG00000002983(Relb)|ENSMUSG00000009406(Elk1)|ENSMUSG00000071076(Jund)|ENSMUSG00000027223(Mapk8ip1)
Oxidative phosphorylation	P17665(Cox7c)	0.41005938917033	0.472459731000598	ENSMUSG00000064341(mt-Nd1)|ENSMUSG00000064356(mt-Atp8)|ENSMUSG00000064360(mt-Nd

				3)|ENSMUSG00000064368(mt-Nd6)|ENSMUSG00000052459(Atp6v1a)|ENSMUSG00000083380(Ndufb4c)|ENSMUSG00000001750(Tcirg1)|ENSMUSG00000033379(Atp6v0b)|ENSMUSG00000030785(Cox6a2)|ENSMUSG00000024038(Ndufv3)|ENSMUSG00000041881(Ndufa7)|ENSMUSG00000033793(Atp6v1h)|ENSMUSG00000025651(Uqcrc1)|ENSMUSG00000020544(Cox11)
Spliceosome	Q9CQ49(Ncbp2)	0.437954637413397	0.493863740061916	ENSMUSG00000021039(Snw1)|ENSMUSG00000038446(Cdc40)|ENSMUSG00000039449(Prpf18)|ENSMUSG00000015656(Hspa8)|ENSMUSG00000020409(Slu7)|ENSMUSG00000091625(Lsm5)|ENSMUSG00000023932(Cdc5l)|ENSMUSG00000029169(Dhx15)|ENSMUSG00000020211(Sf3a2)|ENSMUSG00000020719(Ddx5)
Ribosome	Q9D1N9(Mrpl21)	0.452896498206419	0.500073216769588	ENSMUSG00000024608(Rps14)|ENSMUSG00000079641(Rpl39)|ENSMUSG00000039640(Mrpl12)|ENSMUSG00000090137(Uba52)|ENSMUSG00000039221(Rpl22l1)|ENSMUSG00000090733(Rps27)|ENSMUSG00000050621(Rps27rt)|ENSMUSG00000087412(Gm15501)|ENSMUSG00000062997(Rpl35)
Chemical carcinogenesis - reactive oxygen species	P17665(Cox7c)	0.522028556156945	0.564643132169757	ENSMUSG00000064341(mt-Nd1)|ENSMUSG00000064356(mt-Atp8)|ENSMUSG00000064360(mt-Nd3)|ENSMUSG00000064368(mt-Nd6)|ENSMUSG00000019699(Akt3)|ENSMUSG00000031633(Slc25a4)|ENSMUSG00000046709(Mapk10)|ENSMUSG000

				00044573(Acp1)|ENSMUSG00000083380(Ndufb4c)|ENSMUSG00000030265(Kras)|ENSMUSG00000021936(Mapk8)|ENSMUSG00000030785(Cox6a2)|ENSMUSG00000024038(Ndufv3)|ENSMUSG00000041881(Ndufa7)|ENSMUSG00000025651(Uqcrc1)
Prion disease	P17665(Cox7c)	0.587196978358351	0.622428797059852	ENSMUSG00000064341(mt-Nd1)|ENSMUSG00000064356(mt-Atp8)|ENSMUSG00000064360(mt-Nd3)|ENSMUSG00000064368(mt-Nd6)|ENSMUSG00000031633(Slc25a4)|ENSMUSG00000046709(Mapk10)|ENSMUSG00000031628(Casp3)|ENSMUSG00000083380(Ndufb4c)|ENSMUSG00000015656(Hspa8)|ENSMUSG00000026229(Psmd1)|ENSMUSG00000033220(Rac2)|ENSMUSG00000040714(Klc3)|ENSMUSG00000021936(Mapk8)|ENSMUSG00000030785(Cox6a2)|ENSMUSG00000024038(Ndufv3)|ENSMUSG00000041881(Ndufa7)|ENSMUSG00000025651(Uqcrc1)|ENSMUSG00000026914(Psmd14)
Parkinson disease	P17665(Cox7c)	0.617517479061612	0.641733850789519	ENSMUSG00000064341(mt-Nd1)|ENSMUSG00000064356(mt-Atp8)|ENSMUSG00000064360(mt-Nd3)|ENSMUSG00000064368(mt-Nd6)|ENSMUSG00000031633(Slc25a4)|ENSMUSG00000046709(Mapk10)|ENSMUSG00000031628(Casp3)|ENSMUSG00000072214(Septin5)|ENSMUSG00000083380(Ndufb4c)|ENSMUSG00000026229(Psmd1)|ENSMUSG00000053897(Slc39a8)|ENSMUSG00000040714(Kl

				c3)|ENSMUSG00000021936(Mapk8)|ENSMUSG00000036273(Lrrk2)|ENSMUSG00000030785(Cox6a2)|ENSMUSG00000090137(Uba52)|ENSMUSG00000020178(Adora2a)|ENSMUSG00000000001(Gnai3)|ENSMUSG00000027668(Mfn1)|ENSMUSG00000024038(Ndufv3)|ENSMUSG00000026663(Atf6)|ENSMUSG00000041881(Ndufa7)|ENSMUSG00000025651(Uqcrc1)|ENSMUSG00000026914(Psmd14)

Supplementary Table S8 KEGG Pathway Analysis of Isoverbascoside vs Control Group in Proteomics of Min6 cell

Map.Name	Proteome_ids	Proteome_pvalue	Proteome_FDR	Transcriptome_ids
Cell adhesion molecules	P32507(Nectin2)|Q80Z24(Negr1)|Q9JKF6(Nectin1)	0.0037258251705481	0.111774755116443	ENSMUSG00000028399(Ptprd)
Adherens junction	P32507(Nectin2)|Q9JKF6(Nectin1)	0.120602633833739	0.747090075594184	ENSMUSG00000000600(Krit1)|ENSMUSG00000062312(Erbb2)|ENSMUSG00000044573(Acp1)|ENSMUSG00000000782(Tcf7)|ENSMUSG00000006932(Ctnnb1)
Neurotrophin signaling pathway	P15209(Ntrk2)|Q61144(Psen2)	0.140446918435226	0.747090075594184	ENSMUSG00000025665(Rps6ka6)|ENSMUSG00000030265(Kras)|ENSMUSG00000028698(Pik3r3)|ENSMUSG00000021936(Mapk8)|ENSMUSG00000073433(Arhgdig)

Hedgehog signaling pathway	Q8C4X2(Csnk1g3)	0.152920713813341	0.747090075594184	ENSMUSG00000018395(Kif3a)|ENSMUSG00000045039(Megf8)
Rap1 signaling pathway	G3X9J0(CSipa1l3)|O09110(Map2k3)	0.169288288992112	0.747090075594184	ENSMUSG00000000600(Krit1)|ENSMUSG00000041992(Rapgef5)|ENSMUSG00000030265(Kras)|ENSMUSG00000028698(Pik3r3)|ENSMUSG00000040003(Magi2)|ENSMUSG00000000001(Gnai3)|ENSMUSG00000037533(Rapgef6)|ENSMUSG00000006932(Ctnnb1)|ENSMUSG00000026959(Grin1)|ENSMUSG00000020431(Adcy1)
Herpes simplex virus 1 infection	P32507(Nectin2)|Q9JKF6(Nectin1)	0.17770980053553	0.747090075594184	ENSMUSG00000058900(Rsl1)|ENSMUSG00000055341(Zfp457)|ENSMUSG00000006720(Zfp184)|ENSMUSG00000072915(Gm12258)|ENSMUSG00000032344(Cgas)|ENSMUSG00000092416(Zfp141)|ENSMUSG00000030469(Zfp719)|ENSMUSG00000026896(Ifih1)|ENSMUSG00000044676(Zfp612)|ENSMUSG00000057367(Birc2)|ENSMUSG00000074194(Zfp791)|ENSMUSG00000037640(Zfp60)|ENSMUSG00000060427(Zfp868)|ENSMUSG00000043090(Zfp866)|ENSMUSG00000059475(Zfp426)|ENSMUSG00000028698(Pik3r3)|ENSMUSG00000026029(Casp8)|ENSMUSG00000067942(Zfp160)|ENSMUSG00000028530(Jak1)|ENSMUSG00000087598(Zfp111)|ENSMUSG00000030486(Zfp108)|ENSMUSG00000066829(Zfp810)|ENSM

				USG00000053600(Zfp472)|ENSMUSG00000029605(Oas1b)|ENSMUSG00000039634(Zfp189)|ENSMUSG00000018379(Srsf1)|ENSMUSG00000036898(Zfp157)|ENSMUSG00000058291(Zfp68)
Notch signaling pathway	Q61144(Psen2)	0.220494774115803	0.747090075594184	ENSMUSG00000000708(Kat2b)|ENSMUSG00000021039(Snw1)|ENSMUSG00000029071(Dvl1)|ENSMUSG00000025158(Rfng)
SNARE interactions in vesicular transport	O89116(Vti1a)	0.229704234552101	0.747090075594184	ENSMUSG00000027287(Snap23)|ENSMUSG00000020894(Vamp2)
JAK-STAT signaling pathway	Q9JM05(Pias4)	0.229704234552101	0.747090075594184	ENSMUSG00000028698(Pik3r3)|ENSMUSG00000028530(Jak1)|ENSMUSG00000068758(Il3ra)
Fc epsilon RI signaling pathway	O09110(Map2k3)	0.247802784360711	0.747090075594184	ENSMUSG00000030265(Kras)|ENSMUSG00000028698(Pik3r3)|ENSMUSG00000021936(Mapk8)
NF-kappa B signaling pathway	Q9JM05(Pias4)	0.256694318137675	0.747090075594184	ENSMUSG00000057367(Birc2)|ENSMUSG00000026778(Prkcq)
Ferroptosis	Q75N73(Slc39a14)	0.256694318137675	0.747090075594184	ENSMUSG00000027602(Map1lc3a)|ENSMUSG00000053897(Slc39a8)|ENSMUSG00000056234(Ncoa4)
TGF-beta signaling pathway	Q08639(Tfdp1)	0.265482297789127	0.747090075594184	ENSMUSG00000002603(Tgfb1)|ENSMUSG00000024563(Smad2)|ENSMUSG00000021706(Zfyve16)|ENSMUSG00000027552(E2f5)|ENSMUSG00000021796(Bmpr1a)

Toll-like receptor signaling pathway	O09110(Map2k3)	0.265482297789127	0.747090075594184	ENSMUSG00000029304(Spp1)|ENSMUSG00000028698(Pik3r3)|ENSMUSG00000021936(Mapk8)|ENSMUSG00000026029(Casp8)|ENSMUSG00000028530(Jak1)
Pancreatic secretion	Q99P58(Rab27b)	0.265482297789127	0.747090075594184	ENSMUSG00000032839(Trpc1)|ENSMUSG00000028962(Slc4a2)|ENSMUSG00000041329(Atp1b2)|ENSMUSG00000040907(Atp1a3)|ENSMUSG00000020431(Adcy1)
Inflammatory mediator regulation of TRP channels	O09110(Map2k3)	0.27416791128156	0.747090075594184	ENSMUSG00000026778(Prkcq)|ENSMUSG00000028698(Pik3r3)|ENSMUSG00000021936(Mapk8)|ENSMUSG00000020431(Adcy1)
PPAR signaling pathway	Q60714(Slc27a1)	0.291236724699728	0.747090075594184	ENSMUSG00000039656(Rxrb)|ENSMUSG00000062515(Fabp4)|ENSMUSG00000007783(Cpt1c)
MAPK signaling pathway	O09110(Map2k3)|P15209(Ntrk2)	0.303848774775853	0.747090075594184	ENSMUSG00000025665(Rps6ka6)|ENSMUSG00000002603(Tgfb1)|ENSMUSG00000020882(Cacnb1)|ENSMUSG00000062312(Erbb2)|ENSMUSG00000030265(Kras)|ENSMUSG00000022329(Stk3)|ENSMUSG00000040390(Map3k10)|ENSMUSG00000069806(Cacng7)|ENSMUSG00000021936(Mapk8)|ENSMUSG00000118668(RPS6KA4)|ENSMUSG00000024242(Map4k3)|ENSMUSG00000055723(Rras2)|ENSMUSG00000071076(Jund)|ENSMUSG00000029378(Areg)|ENSMUSG00000027223(Mapk8ip1)

Lysine degradation	O08550	0.307909996380139	0.747090075594184	ENSMUSG00000071350(Setdb2)|ENSMUSG00000029687(Ezh2)|ENSMUSG00000026646(Suv39h2)|ENSMUSG00000013787(Ehmt2)|ENSMUSG00000010025(Aldh3a2)
GnRH signaling pathway	O09110(Map2k3)	0.324196756649536	0.747090075594184	ENSMUSG00000030265(Kras)|ENSMUSG00000021936(Mapk8)|ENSMUSG00000020431(Adcy1)
PD-L1 expression and PD-1 checkpoint pathway in cancer	O09110(Map2k3)	0.324196756649536	0.747090075594184	ENSMUSG00000030265(Kras)|ENSMUSG00000021109(Hif1a)|ENSMUSG00000026778(Prkcq)|ENSMUSG00000028698(Pik3r3)|ENSMUSG00000028530(Jak1)
Toxoplasmosis	O09110(Map2k3)	0.332197961333135	0.747090075594184	ENSMUSG00000002603(Tgfb1)|ENSMUSG00000057367(Birc2)|ENSMUSG00000046879(Irgm1)|ENSMUSG00000021936(Mapk8)|ENSMUSG00000026029(Casp8)|ENSMUSG00000028530(Jak1)|ENSMUSG00000000001(Gnai3)
TNF signaling pathway	O09110(Map2k3)	0.340105832770158	0.747090075594184	ENSMUSG00000052837(Junb)|ENSMUSG00000057367(Birc2)|ENSMUSG00000028698(Pik3r3)|ENSMUSG00000021936(Mapk8)|ENSMUSG00000026029(Casp8)|ENSMUSG00000118668(RPS6KA4)|ENSMUSG00000056501(Cebpb)
Polycomb repressive complex	Q08639(Tfdp1)	0.36328011122933	0.747090075594184	ENSMUSG00000029687(Ezh2)|ENSMUSG00000025409(Mbd6)|ENSMUSG00000025616(Usp16)|ENSMUSG00000030619(Eed)|ENSMUSG00000029836(Cbx3)|ENSMUSG00000000037(S

				cml2)|ENSMUSG00000028345(Tex10)
Glycerophospholipid metabolism	Q8BSF4(Pisd)	0.385652276637298	0.747090075594184	ENSMUSG00000040774(Cept1)|ENSMUSG00000040479(Dgkz)
Calcium signaling pathway	P15209(Ntrk2)	0.400134982829959	0.747090075594184	ENSMUSG00000062312(Erbb2)|ENSMUSG00000049686(Orai1)|ENSMUSG00000023990(Tfeb)|ENSMUSG00000026959(Grin1)|ENSMUSG00000020431(Adcy1)
Growth hormone synthesis, secretion and action	O09110(Map2k3)	0.400134982829959	0.747090075594184	ENSMUSG00000030265(Kras)|ENSMUSG00000052837(Junb)|ENSMUSG00000028698(Pik3r3)|ENSMUSG00000021936(Mapk8)|ENSMUSG00000000001(Gnai3)|ENSMUSG00000020431(Adcy1)
Alcoholic liver disease	O09110(Map2k3)	0.434886620188237	0.747090075594184	ENSMUSG00000022858(Tra2b)|ENSMUSG00000010025(Aldh3a2)|ENSMUSG00000021936(Mapk8)|ENSMUSG00000007783(Cpt1c)|ENSMUSG00000026029(Casp8)|ENSMUSG00000030281(Il17rc)|ENSMUSG00000000782(Tcf7)|ENSMUSG00000006932(Ctnnb1)
Alcoholism	P15209(Ntrk2)	0.467653334885029	0.747090075594184	ENSMUSG00000024891(Slc29a2)|ENSMUSG00000030265(Kras)|ENSMUSG00000003545(Fosb)|ENSMUSG00000027018(Hat1)|ENSMUSG00000000001(Gnai3)|ENSMUSG00000026959(Grin1)

Insulin resistance	Q60714(Slc27a1)	0.473978432697177	0.747090075594184	ENSMUSG00000025665(Rps6ka6)|ENSMUSG00000026778(Prkcq)|ENSMUSG00000028698(Pik3r3)|ENSMUSG00000021936(Mapk8)|ENSMUSG00000039515(Ptpa)
Fluid shear stress and atherosclerosis	Q9JM05(Pias4)	0.473978432697177	0.747090075594184	ENSMUSG00000021270(Hsp90aa1)|ENSMUSG00000020048(Hsp90b1)|ENSMUSG00000028698(Pik3r3)|ENSMUSG00000021936(Mapk8)|ENSMUSG00000021796(Bmpr1a)|ENSMUSG00000006932(Ctnnb1)|ENSMUSG00000020738(Sumo2)
Thyroid hormone signaling pathway	Q9DB40(Med27)	0.486407352036495	0.747090075594184	ENSMUSG00000039656(Rxrb)|ENSMUSG00000000708(Kat2b)|ENSMUSG00000030265(Kras)|ENSMUSG00000021109(Hif1a)|ENSMUSG00000028698(Pik3r3)|ENSMUSG00000041329(Atp1b2)|ENSMUSG00000040907(Atp1a3)|ENSMUSG00000006932(Ctnnb1)
Hepatitis B	O09110(Map2k3)	0.504510202413827	0.747090075594184	ENSMUSG00000002603(Tgfb1)|ENSMUSG00000026896(Ifih1)|ENSMUSG00000030265(Kras)|ENSMUSG00000028698(Pik3r3)|ENSMUSG00000021936(Mapk8)|ENSMUSG00000026029(Casp8)|ENSMUSG00000000787(Ddx3x)|ENSMUSG00000028530(Jak1)
MicroRNAs in cancer	P70345(Bcl2l2)	0.516228042989925	0.747090075594184	ENSMUSG00000029687(Ezh2)|ENSMUSG00000032050(Rdx)|ENSMUSG00000062312(Erbb2)|ENSMUSG00000030265(Kras)|ENSMUSG00000020326(Ccng1)|ENSMUSG00000024975(

				Pdcd4)|ENSMUSG00000028698(Pik3r3)|ENSMUSG00000032254(Kif23)|ENSMUSG00000020135(Apc2)
Cellular senescence	O09110(Map2k3)	0.521984164659198	0.747090075594184	ENSMUSG00000002603(Tgfb1)|ENSMUSG00000032113(Chek1)|ENSMUSG00000030265(Kras)|ENSMUSG00000024563(Smad2)|ENSMUSG00000032409(Atr)|ENSMUSG00000028698(Pik3r3)|ENSMUSG00000027552(E2f5)|ENSMUSG00000055723(Rras2)|ENSMUSG00000028224(Nbn)
Yersinia infection	O09110(Map2k3)	0.527672807815118	0.747090075594184	ENSMUSG00000025665(Rps6ka6)|ENSMUSG00000057672(Pkn1)|ENSMUSG00000026341(Actr3)|ENSMUSG00000028698(Pik3r3)|ENSMUSG00000021936(Mapk8)|ENSMUSG00000020152(Actr2)
mRNA surveillance pathway	Q9CQ49(Ncbp2)	0.538850766516547	0.747090075594184	ENSMUSG00000036572(Upf3b)|ENSMUSG00000024360(Etf1)|ENSMUSG00000041459(Tardbp)|ENSMUSG00000020273(Papolg)|ENSMUSG00000043241(Upf2)|ENSMUSG00000021111(Papola)|ENSMUSG00000042271(Nxt2)|ENSMUSG00000030655(Smg1)
Lipid and atherosclerosis	O09110(Map2k3)	0.570844394296737	0.747090075594184	ENSMUSG00000021270(Hsp90aa1)|ENSMUSG00000024924(Vldlr)|ENSMUSG00000039656(Rxrb)|ENSMUSG00000020048(Hsp90b1)|ENSMUSG00000030265(Kras)|ENSMUSG00000025980(Hspd1)|ENSMUSG00000028698(Pik3r3)|

				ENSMUSG00000021936(Mapk8)|ENSMUSG00000026029(Casp8)
mTOR signaling pathway	Q8C0M0(Wdr59)	0.575959751987916	0.747090075594184	ENSMUSG00000052459(Atp6v1a)|ENSMUSG00000025665(Rps6ka6)|ENSMUSG00000030265(Kras)|ENSMUSG00000011096(Akt1s1)|ENSMUSG00000028698(Pik3r3)|ENSMUSG00000070934(Rraga)|ENSMUSG00000029071(Dvl1)
Pathways of neurodegeneration - multiple diseases	O09110(Map2k3)|O70305(Atxn2)|Q61144(Psen2)	0.578172724369203	0.747090075594184	ENSMUSG00000064368(mt-Nd6)|ENSMUSG00000017831(Rab5a)|ENSMUSG00000032965(Ift57)|ENSMUSG00000041459(Tardbp)|ENSMUSG00000083380(Ndufb4c)|ENSMUSG00000030265(Kras)|ENSMUSG00000040390(Map3k10)|ENSMUSG00000027602(Map1lc3a)|ENSMUSG00000040714(Klc3)|ENSMUSG00000021936(Mapk8)|ENSMUSG00000026029(Casp8)|ENSMUSG00000029071(Dvl1)|ENSMUSG00000025889(Snca)|ENSMUSG00000036273(Lrrk2)|ENSMUSG00000090137(Uba52)|ENSMUSG00000027668(Mfn1)|ENSMUSG00000020135(Apc2)|ENSMUSG00000006932(Ctnnb1)|ENSMUSG00000025907(Rb1cc1)|ENSMUSG00000026959(Grin1)|ENSMUSG00000026914(Psmd14)

Ras signaling pathway	P15209(Ntrk2)	0.586010941508315	0.747090075594184	ENSMUSG00000017831(Rab5a)|ENSMUSG00000041992(Rapgef5)|ENSMUSG00000030265(Kras)|ENSMUSG00000028698(Pik3r3)|ENSMUSG00000024976(Shoc2)|ENSMUSG00000021936(Mapk8)|ENSMUSG00000067629(Syngap1)|ENSMUSG00000055723(Rras2)|ENSMUSG00000026959(Grin1)
Ubiquitin mediated proteolysis	Q9JM05(Pias4)	0.590948155271032	0.747090075594184	ENSMUSG00000032030(Cul5)|ENSMUSG00000032307(Ube2q2)|ENSMUSG00000026219(Trip12)|ENSMUSG00000057367(Birc2)|ENSMUSG00000030061(Uba3)|ENSMUSG00000024231(Cul2)|ENSMUSG00000040782(Cop1)|ENSMUSG00000035898(Uba6)|ENSMUSG00000021774(Ube2e1)|ENSMUSG00000090137(Uba52)|ENSMUSG00000029176(Anapc4)|ENSMUSG00000020687(Cdc27)
Biosynthesis of cofactors	Q9R1Z7(Pts)	0.600649216792855	0.747090075594184	ENSMUSG00000010025(Aldh3a2)|ENSMUSG00000026615(Eprs)|ENSMUSG00000042642(Flad1)|ENSMUSG00000001891(Ugp2)
Epstein-Barr virus infection	O09110(Map2k3)	0.600649216792855	0.747090075594184	ENSMUSG00000021039(Snw1)|ENSMUSG00000028698(Pik3r3)|ENSMUSG00000021936(Mapk8)|ENSMUSG00000026029(Casp8)|ENSMUSG00000028530(Jak1)|ENSMUSG00000029605(Oas1b)|ENSMUSG00000026914(Psmd14)

Lysosome	Q69ZN6(Gnptab)	0.605414399780366	0.747090075594184	ENSMUSG00000028015(Ctso)|ENSMUSG00000030720(Cln3)|ENSMUSG00000022066(Entpd4b)|ENSMUSG00000002204(Napsa)|ENSMUSG00000021824(Ap3m1)|ENSMUSG00000001750(Tcirg1)|ENSMUSG00000047126(Cltc)
Spinocerebellar ataxia	O70305(Atxn2)	0.61012356173525	0.747090075594184	ENSMUSG00000024924(Vldlr)|ENSMUSG00000062785(Kcnc3)|ENSMUSG00000028698(Pik3r3)|ENSMUSG00000021936(Mapk8)|ENSMUSG00000025907(Rb1cc1)|ENSMUSG00000026959(Grin1)|ENSMUSG00000026914(Psmd14)
Nucleocytoplasmic transport	Q9CQ49(Ncbp2)	0.641572678713119	0.759831077499311	ENSMUSG00000027787(Nmd3)|ENSMUSG00000036572(Upf3b)|ENSMUSG00000021929(Kpna3)|ENSMUSG00000026491(Ahctf1)|ENSMUSG00000043241(Upf2)|ENSMUSG00000003226(Ranbp2)|ENSMUSG00000042271(Nxt2)|ENSMUSG00000024287(Thoc1)|ENSMUSG00000066232(Ipo7)|ENSMUSG00000012535(Tnpo3)|ENSMUSG00000053453(Thoc7)|ENSMUSG00000034826(Nup54)|ENSMUSG00000006005(Tpr)|ENSMUSG00000020738(Sumo2)|ENSMUSG00000002718(Cse1l)
Cell cycle	Q08639(Tfdp1)	0.645856415874414	0.759831077499311	ENSMUSG00000023908(Pkmyt1)|ENSMUSG00000022141(Nipbl)|ENSMUSG00000002603(Tgfb1)|ENSMUSG00000032113(Chek1)|ENSMUSG00000038379(Ttk)|ENSMUSG000000245

				63(Smad2)|ENSMUSG00000002297(Dbf4)|ENSMUSG00000032409(Atr)|ENSMUSG00000024056(Ndc80)|ENSMUSG00000024974(Smc3)|ENSMUSG00000026037(Orc2)|ENSMUSG00000027552(E2f5)|ENSMUSG00000029176(Anapc4)|ENSMUSG00000020687(Cdc27)|ENSMUSG00000040044(Orc3)|ENSMUSG00000027379(Bub1)|ENSMUSG00000027326(Knl1)
PI3K-Akt signaling pathway	P15209(Ntrk2)	0.662492763211665	0.76441472678269	ENSMUSG00000021270(Hsp90aa1)|ENSMUSG00000020048(Hsp90b1)|ENSMUSG00000057672(Pkn1)|ENSMUSG00000062312(Erbb2)|ENSMUSG00000029304(Spp1)|ENSMUSG00000030265(Kras)|ENSMUSG00000028698(Pik3r3)|ENSMUSG00000040003(Magi2)|ENSMUSG00000028530(Jak1)|ENSMUSG00000068758(Il3ra)|ENSMUSG00000026785(Pkn3)|ENSMUSG00000028145(Them4)|ENSMUSG00000029378(Areg)|ENSMUSG00000001506(Col1a1)
Human immunodeficiency virus 1 infection	O09110(Map2k3)	0.686013850123134	0.776619452969586	ENSMUSG00000032344(Cgas)|ENSMUSG00000032113(Chek1)|ENSMUSG00000032030(Cul5)|ENSMUSG00000030265(Kras)|ENSMUSG00000032409(Atr)|ENSMUSG00000028698(Pik3r3)|ENSMUSG00000021936(Mapk8)|ENSMUSG00000026029(Casp8)|ENSMUSG00000000001(Gnai3)

Alzheimer disease	Q61144(Psen2)|Q75N73(Slc39a14)	0.719387091817712	0.790927487273267	ENSMUSG00000064368(mt-Nd6)|ENSMUSG00000083380(Ndufb4c)|ENSMUSG00000030265(Kras)|ENSMUSG00000028698(Pik3r3)|ENSMUSG00000053897(Slc39a8)|ENSMUSG00000040714(Klc3)|ENSMUSG00000021936(Mapk8)|ENSMUSG00000026029(Casp8)|ENSMUSG00000029071(Dvl1)|ENSMUSG00000025889(Snca)|ENSMUSG00000031878(Nae1)|ENSMUSG00000020135(Apc2)|ENSMUSG00000006932(Ctnnb1)|ENSMUSG00000025907(Rb1cc1)|ENSMUSG00000026959(Grin1)|ENSMUSG00000026914(Psmd14)
Spliceosome	Q9CQ49(Ncbp2)	0.725016863333828	0.790927487273267	ENSMUSG00000021039(Snw1)|ENSMUSG00000021500(Ddx46)|ENSMUSG00000022858(Tra2b)|ENSMUSG00000025982(Sf3b1)|ENSMUSG00000024287(Thoc1)|ENSMUSG00000023932(Cdc5l)|ENSMUSG00000029169(Dhx15)|ENSMUSG00000020211(Sf3a2)|ENSMUSG00000020719(Ddx5)|ENSMUSG00000018379(Srsf1)
Ribosome	Q9D1N9(Mrpl21)	0.741128355285424	0.794066094948669	ENSMUSG00000052146(Rps10)|ENSMUSG00000090137(Uba52)|ENSMUSG00000050621(Rps27rt)|ENSMUSG00000029486(Mrpl1)
Amyotrophic lateral sclerosis	O09110(Map2k3)|O70305(Atxn2)	0.775440629130591	0.816253293821674	ENSMUSG00000064368(mt-Nd6)|ENSMUSG00000017831(Rab5a)|ENSMUSG00000041459(Tardbp)|ENSMUSG00000083380(Ndufb4c)|ENSMUSG00000004980(Hnrnpa2b1)|ENSMUS

				G00000003226(Ranbp2)|ENSMUSG00000042271(Nxt2)|ENSMUSG00000027602(Map1lc3a)|ENSMUSG00000040714(Klc3)|ENSMUSG00000034826(Nup54)|ENSMUSG00000025907(Rb1cc1)|ENSMUSG00000006005(Tpr)|ENSMUSG00000026959(Grin1)|ENSMUSG00000026914(Psmd14)
Salmonella infection	O09110(Map2k3)	0.815581131360119	0.83166818457732	ENSMUSG00000021270(Hsp90aa1)|ENSMUSG00000021929(Kpna3)|ENSMUSG00000020048(Hsp90b1)|ENSMUSG00000017831(Rab5a)|ENSMUSG00000057672(Pkn1)|ENSMUSG00000057367(Birc2)|ENSMUSG00000043463(Rab9b)|ENSMUSG00000026341(Actr3)|ENSMUSG00000040714(Klc3)|ENSMUSG00000021936(Mapk8)|ENSMUSG00000026029(Casp8)|ENSMUSG00000079316(Rab9)|ENSMUSG00000031176(Dynlt3)|ENSMUSG00000000782(Tcf7)|ENSMUSG00000029763(Exoc4)|ENSMUSG00000030660(Pik3c2a)|ENSMUSG00000034247(Plekhm1)|ENSMUSG00000006932(Ctnnb1)|ENSMUSG00000027002(Nckap1)|ENSMUSG00000002718(Cse1l)|ENSMUSG00000020152(Actr2)
Thermogenesis	O09110(Map2k3)	0.817807048167698	0.83166818457732	ENSMUSG00000064368(mt-Nd6)|ENSMUSG00000025665(Rps6ka6)|ENSMUSG00000053470(Kdm3a)|ENSMUSG00000083380(Ndufb4c)|

				ENSMUSG00000030265(Kras)|ENSMUSG00000011096(Akt1s1)|ENSMUSG00000007783(Cpt1c)|ENSMUSG00000020431(Adcy1)
Parkinson disease	Q75N73(Slc39a14)	0.883928220350592	0.883928220350592	ENSMUSG00000064368(mt-Nd6)|ENSMUSG00000083380(Ndufb4c)|ENSMUSG00000053897(Slc39a8)|ENSMUSG00000040714(Klc3)|ENSMUSG00000021936(Mapk8)|ENSMUSG00000025889(Snca)|ENSMUSG00000036273(Lrrk2)|ENSMUSG00000090137(Uba52)|ENSMUSG00000000001(Gnai3)|ENSMUSG00000027668(Mfn1)|ENSMUSG00000026914(Psmd14)
